# Morphometric analysis of Eocene nummulitids in western and central Cuba: taxonomy, biostratigraphy and evolutionary trends

**DOI:** 10.1080/14772019.2018.1446462

**Published:** 2018-04-13

**Authors:** Ana. I. Torres-Silva, Wolfgang Eder, Johann Hohenegger, Antonino Briguglio

**Affiliations:** a Department of Palaeontology, University of Vienna, Althanstrasse 14, 1090, Vienna, Austria; b Dipartimento di Scienze della, Terra dell'Ambiente e della Vita, Universitá degli Studi di Genova, Corso Europa, 26, I-16132Genova, Italy

**Keywords:** Nummulitidae, larger foraminifera, morphometry, growth-independent and growth-invariant characters, Eocene

## Abstract

Megalospheric specimens of Nummulitidae from eight localities in western and central Cuba were morphometrically investigated using test characters described by 11 growth-independent and growth-invariant attributes that provide a complete geometric reconstruction of nummulitid equatorial morphology. The species *Nummulites striatoreticulatus*, *Palaeonummulites trinitatensis*, *Operculinoides floridensis* and *O. soldadensis* were classified by an agglomerative cluster analysis. Discriminant analysis yielded significant morphological separators between the species such as the backbend angle, marginal radius increase, perimeter ratio and first chamber length. The transition of tightness to laxity of the spiral was an important morphological separator at the generic level, representing a clear general trend coupled with the change in palaeodepth. Based on further discriminant analysis, an increase in proloculus size was detected in *Nummulites striatoreticulatus* from the middle Eocene to early late Eocene, supporting this important evolutionary pattern in many lineages of *Nummulites*. Operculinid forms showed an opposite and more weakly pronounced time-dependent trend in the size decrease of the proloculus. In the Cuban localities, *Nummulites striatoreticulatus* occurs from the Lutetian to Priabonian, while *Palaeonummulites trinitatensis* is restricted to the Bartonian to Priabonian. The moderately to loosely coiled operculinid taxa *O. floridensis* and *O. soldadensis* have longer stratigraphical ranges from the middle Eocene to probably the early Oligocene. *Operculinoides floridensis* and *O. soldadensis* show a broader variability in marginal radius increase, and thus probably occupied wider niches than *N. striatoreticulatus*. The latter seems to be restricted to the shelf edge and to the shallowest parts of the upper slope. A possible phylogenetic connection between *Heterostegina* and *Operculinoides* is suggested by the closest equatorial morphology of *Heterostegina* sp. indet. to tightly coiled forms of *Operculinoides floridensis*. Discriminant analysis documents the strongest similarities in perimeter ratio, backbend angle, initial marginal radius and proloculus mean diameter.

## Introduction

Among the symbiont-bearing larger benthic foraminifera (LBF), nummulitids are one of the most common and widespread groups in shallow-marine, warm-temperate to tropical carbonate environments throughout the Cenozoic. Especially in the Eocene, nummulitid communities around the Tethyan, Indo-Pacific and American-Caribbean provinces achieved their highest abundances combined with high evolutionary rates. Worldwide, they document the maturity and evolution of benthic communities better than other LBF groups (Cole 1958, 1964; Frost & Langenheim 1974; Hottinger 1977; Schaub 1981; Serra-Kiel *et al*. 1998; Tosquella & Serra-Kiel 1998; Less *et al*. 2008; Haynes *et al*. 2010; BouDagher-Fadel & Price 2014; Lunt & Renema 2014; Benedetti *et al*. 2017; Torres-Silva *et al*. 2017). Nummulitidae de Blainville, 1827 belong to the lamellar-perforate LBF with planispiral enrolment, which can be approximated by a logarithmic spiral. Their hyaline tests range from involute to evolute. A marginal cord with an internal canal system is always present, as is an initial embryonic apparatus consisting of a proloculus and deuteroloculus. This embryonic part is followed by numerous equatorial chambers which may be undivided (e.g. *Nummulites*, *Palaeonummulites*, *Assilina*, *Ranikothalia*, *Operculinella*, *Operculina*) or divided into chamberlets by secondary septa (e.g. *Planoperculina*, *Planostegina*, *Heterostegina*, *Spiroclypeus*, *Cycloclypeus*).

Nummulitids without chamber partitions, although stratigraphically useful, are difficult to assign to generic or specific level. The variability of their features and the abundance of transitional forms has proved to be so strong that Cole in the *Treatise on invertebrate paleontology* (Loeblich & Tappan 1964) considered characters such as the degree of involution, number of whorls, whorl height, chamber shape and spiral development to define species rather than genera. Accordingly, *Palaeonummulites*, *Operculinoides*, *Ranikothalia*, *Assilina* and *Operculina* were placed into synonymy with *Nummulites* ( = *Camerina*). Later studies in the Tethyan province clarified this unsatisfactory taxonomic situation. Delimitations, as well as details of evolutionary transitions within species, are well investigated today (Hottinger 1977; Schaub 1981; Haynes *et al*. 2010). Compared to the Tethyan province, intraspecific evolution in the Caribbean remains understudied and generic nomenclature has not yet reached any consensus. The great range of nummulitid morphology included by some authors in a single genus and/or species (Cole in Loeblich & Tappan 1964; Frost & Langenheim 1974; Butterlin 1981) has obscured the possible existence of closely related genera/or species with overlapping morphological variations. On the one hand, taxa similar to *Nummulites* sensu stricto with involute tests, tight coiling with numerous whorls, and rather equidimensional chambers, present few difficulties for generic classification. On the other, there is much confusion about the assignment of small, involute to semi-involute forms with rapidly widening coils and simple primary septa. These forms show intermediate features between *Nummulites* and *Operculina*, and have therefore been placed in *Nummulites* Lamarck, *Palaeonummulites* Schubert, *Caudriana* Haynes, *Operculinoides* Hanzawa, *Operculina* d'Orbigny or *Operculinella* Yabe (Barker 1939; Cole 1958, 1960, 1964; Nagappa 1959; Eames *et al*. 1962; Frost & Langenheim 1974; Robinson & Wright 1993; Mello e Sousa *et al*. 2003; Robinson 2004; Haynes *et al.* 2010; BouDagher-Fadel & Price 2014; Molina *et al*. 2016). Quantification of test morphology appears to be the most appropriate method to solve the present taxonomic problems, and to reduce the degree of subjectivity inherent in traditional taxonomic studies based on morphology.

Nummulitids without chamber partitions have been previously morphometrically investigated in the Caribbean province to attempt generic or specific delimitation (Wright & Switzer 1971; Barnett 1974; Frost & Langenheim 1974; Bowen-Powell 2010). Nonetheless, nummulitid tests have been often characterized by a small set of measurements (e.g. test diameter, proloculus diameter, chamber number per whorl, whorl diameters), which do not provide complete test reconstruction and only allow comparison between individuals at similar growth stages (Hohenegger 2011b). This issue becomes even more complex by including life cycles and morphological responses to environmental conditions. In the extant nummulitid *Heterostegina depressa* it has recently been demonstrated that proloculus size and the number of operculine chambers exhibit a strong variability because populations consist of a mix of two megalospheric morphotypes (Eder *et al*. 2017a). Each growth step, represented by the addition of a single chamber, marks the response of the growing cell to its environment by size and shape (Hohenegger 2011b; Ferrández-Cañadell 2012; Briguglio *et al*. 2013; Renema & Cotton 2015). Loosely coiled nummulitids of the Caribbean Eocene vary in size and shape depending on the depositional environment (Cole 1958). Thus, the biology of LBF reflects their growth strategies, their environmental conditions and their morphological adaptation to the environment. In the fossil record, where molecular investigations remain impossible, species delimitation based on morphology has to be treated simultaneously using a multitude of morphological characters to explain test shapes dependent on niches and evolutionary tendencies (Hohenegger 2014). Morphological quantification based on growth-independent and growth-invariant characters has proved to be an adequate tool to explain the complete change in test shape during ontogeny, to clarify phylogenetic relations and to define morphospecies in fossil forms (Hohenegger 2011b; Eder *et al*. 2017b; Hohenegger &Torres-Silva 2017). Quantification of test morphology in the fossil record using growth-independent and growth-invariant characters was carried out for the first time on *Heterostegina* from the Cuban Eocene (Torres-Silva *et al*. 2017). This study allowed enhanced species recognition and better interpretation of evolutionary trends separated from environmental and palaeogeographical diversification.

Otherwise, despite the abundance of Nummulitidae in the Cuban Palaeogene, taxonomic studies are limited. Nummulitid assemblages have been published without illustrations (Bermúdez 1950; Brönnimann & Rigassi 1963; Blanco-Bustamante *et al*. 1987; García-Delgado & Torres-Silva 1997; Torres-Silva *et al*. 2001) or reported as part of systematic geological mapping of Cuba, but the bulk of this information has remained unpublished. Only a few taxonomic works are available on Cuban nummulitids (Palmer 1934; Rutten 1935; Cizancourt 1947; Montero 1981). Thus, the diversity, evolutionary trends and biostratigraphical ranges in this group remain poorly known. Moreover, in the Caribbean province data on their morphological responses to ecological gradients is sparse.

This study focuses on the morphology of Eocene Nummulitinae from eight localities in western and central Cuba, spanning the time interval from middle Eocene to lower Oligocene. We use growth-independent and growth-invariant characters (Hohenegger & Torres-Silva 2017) to describe the internal morphology of megalospheric individuals (A forms) because B forms (microspheres) are rare. The research was designed to investigate intraspecific variation, stratigraphical ranges and evolutionary trends. In addition, it deals with the relationship between different palaeoenvironments and the variability in test morphology. Morphological and ecological observations, particularly those related to extant nummulitids (Hohenegger 1999; Beavington-Penney & Racey 2004; Yordanova & Hohenegger 2004; Eder *et al*. 2017a, b), are integrated within the context of the ecology of fossil LBF. Finally, we discuss the phylogenetic connections between the nummulitid species described here and the *Heterostegina* species reported by Torres-Silva *et al*. (2017).

## Geological setting

**Figure 1.**
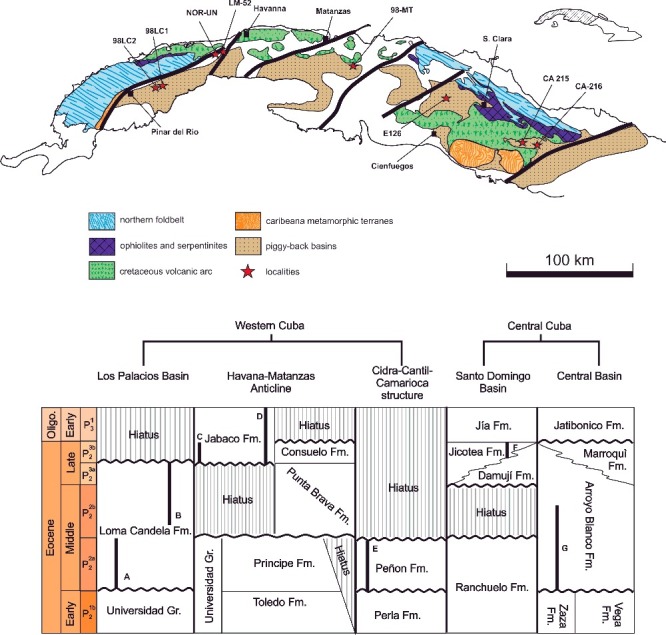
**A,** schematic tectonic map of western and central Cuba (after Iturrlade-Vinent 1994), with locations of the stratigraphical sections and samples. **B,** stratigraphical relations of Eocene units in western and central Cuba, slightly modified from García-Delgado & Torres-Silva (1997); stratigraphical ranges of the studied sections: A, 98LC-2; B, 98LC-1; C, LM-52; D, NOR-UN; E, 98MT-1; F, E-126; G, CA-215.

The termination of the collision process between the North American Plate (NOAM) and the Cuban segment of the Greater Antillean Cretaceous Arc (GAKA) started in western Cuba between the latest Paleocene and early Eocene (Bralower & Iturralde-Vinent 1997) and shifted towards central and eastern Cuba in the middle to late Eocene (Gordon *et al*. 1997). This ongoing tectonic scenario led to the emplacement not only of the major Cuban foldbelt but also of north-east- to east-trending strike-slip faults (Pinar, Matanzas, La Trocha, Cauto Faults) and related piggybacks formed to the south of the major faults on the allochthonous thrust units of the extinct the Cretaceous volcanic arc and ophiolites (Fig. 1A). These basins, structurally separated from each another, occur across Cuba and divide the island into tectonostratigraphical units. They became sites for the deposition of syn- and post-orogenic Maastrichtian to Eocene sediments, and the unconformably overlying neoplatformic stage (neo-autochthonous) from the latest Eocene to Quaternary (Iturralde-Vinent 1994).

The current investigation includes six sections and two sample sites of the Loma Candela, Jabaco, Peñon, Jicotea, Arroyo Blanco and Blanco formations that reflect, starting from the middle Eocene, the post-orogenic history of these strike-slip basins in western and central Cuba (Iturralde-Vinent 1995; Garcia-Delgado & Torres-Silva 1997) (Fig. 1A).

### Western Cuba

#### Loma Candela Formation

Deposits of the Loma Candela Formation (Bermúdez 1950) are part of the Los Palacios Basin, a strike-slip and piggyback basin (Iturralde-Vinent 1995, 1996; García-Delgado & Torres-Silva 1997) south of the Pinar Fault on top of the GAKA. Outcrops of this unit are rare and exist only in a narrow and discontinuous belt south of the Guaniguanico Mountain Range around San Diego de los Baños (Pinar del Rio province). According to García-Delgado &Torres-Silva 1997), the early Eocene Capdevila Formation and the Universidad Group are topped by an erosional unconformity covered by the transgressive Loma Candela Formation. The Oligocene–Miocene Paso Real Formation, unconformably overlies this unit (Fig. 1B). It comprises mainly conglomerates interbedded with marls and marly limestones with abundant LBF, echinoids, bivalves and gastropods.

The investigated nummulitid specimens from the Loma Candela Formation come from the Entronque de Herradura and Loma Candelaria localities. The basal transgressive part of the Loma Candela Formation is well exposed at a quarry (site label 98LC-2) located near Entronque de Herradura in Pinar del Río province (22°30′56″N; 83°5′39″W) (Fig. 1A). A massive succession of yellowish limestones and marly limestones, about 8 m thick, contains mainly larger benthic foraminifera. Nummulitids accompanied by bivalves, echinoids and gastropods dominate the fossil fauna.

The upper part of Loma Candela Formation crops out at Loma Candelaria (site label 98LC-1) along the roadside to San Diego de los Baños (22°35.931′N; 83°23.130′W) (Fig. 1A). At this locality, it is partially exposed as a nearly 20 m thick sequence of conglomerates, interbedded with limestones and marls bearing mainly nummulitids. Larger benthic foraminiferal assemblages and calcareous nannofossils were studied by Torres-Silva *et al*. (2017) and assigned to zones NP 16/17 corresponding to Bartonian to early Priabonian.

#### Jabaco Formation

The Jabaco Formation forms part of the infill of the Havana-Matanzas Anticline, a sedimentary record similar to Los Palacios basin, which also rests over the Cretaceous volcanic arc in Havana and the western part of Matanzas provinces (Àlbear-Franquiz & Iturralde-Vinent 1985; García-Delgado & Torres-Silva 1997). Bermúdez (1937) introduced the Jabaco Formation as comprising the hemipelagic marls and interbedded argillaceous limestones developed as a narrow belt at Loma Jabaco west of Havana province. In Loma Jabaco, this unit rests on an erosional unconformity above the early Eocene Capdevila Formation and the Universidad Group, unconformably covered by the Miocene Cojimar and Oligocene Guanajay formations (García-Delgado & Torres-Silva 1997; Fig. 1B). The first biostratigraphical studies were carried out by Bermúdez (1937, 1950) and Brönninamn & Rigassi (1963), mainly using smaller benthic and planktonic foraminifera. The latter authors assigned the Jabaco Formation to the *Turborotalia cerroazulensis* zone corresponding to late Eocene.

The investigated nummulitid specimens from the Jabaco Formation come from the localities of Loma Jabaco and Noroña. At Loma Jabaco (site label LM-52), a sample from a barely exposed sequence of hemipelagic marls with intercalated argillaceous limestones was studied, outcropping near the intersection of the road from Guanajay to El Mariel, 4.5 km W–NW of Guanajay (Havana province, western Cuba; 22°56.647′N; 82°43.809′W; Fig. 1A). Torres-Silva *et al*. (2017) studied the LBF and calcareous nannofossil assemblages and attributed this locality according to Martini (1971) to the calcareous nannofossil zone NP 19–20, corresponding to late Priabonian. The Noroña section (site label NOR-UN) is exposed as nearly 50 m of hemipelagic marls with intercalated argillaceous limestones and occasional sandstones beds. This section, including the Eocene–Oligocene (E/O) boundary, is located near Guanajay (22°57′22.907″N; 82°41′43.023″W) (Fig. 1A). The LBF assemblages were positioned above the E/O boundary in the lower and middle part of planktonic foram zone O1 (P18) and in the middle part of calcareous nannofossil zone NP 21 (CP 16), where both plankton zones suggest a Rupelian age (Molina *et al*. 2016).

#### Peñon Formation

The lowest middle Eocene shallow carbonate unit exposed in western Cuba is the Peñon Formation (Brödermann 1945). This unit infills the central and northern regions of the Cidra-Cantil-Camarioca Structure in an area of low topographic relief. It is intensively covered by residual soils; thus, outcrops are scarce. The Peñon Formation rests unconformably on the Cretaceous Peñalver, Via Blanca and Chirino formations, as well as the early Eocene Perla Formation, and is unconformably overlain by the Miocene Arabos and Güines units (García-Delgado & Torres-Silva 1997) (Fig. 1B). Cole & Gravell (1952) dated the type locality of the Peñon Formation as early middle Eocene, based mostly on the occurrences of helicosteginids and orthophragminids. The absence of nummulitids was also noted and attributed to ecological conditions and/or may reflect the somewhat older age of this locality. Quite close to the type locality, at Angelita Quarry, a similar LBF assemblage with nummulitid occurrences was reported by Torres-Silva *et al*. (2001). Although no isolated nummulitids could be investigated from this locality, their occurrences are included here for biostratigraphical and palaeoecological purposes. At the Angelita Quarry (site label 98MT-1) a section was logged north of Anguila village, Martí township, Matanzas Province (22°56′51″N; 80°55′43.13″W) (Fig. 1A). It consists of a roughly 2 m long sequence of grey calcareous sandstones intercalated with calcarenites, which are infiltrated by heavy bitumen.

### Central Cuba

#### Jicotea Formation

The Jicotea Formation (Bermúdez 1950) represents the post-orogenic late Eocene sediments, which are irregularly exposed NE of the Santo Domingo Basin, a piggyback basin located westwards of the Las Villas block and related to the La Trocha fault (García-Delgado & Torres-Silva 1997). The Jicotea Formation comprises a marly and calcareous series of marls to mudstones, polymictic sandstones, conglomerates, calcarenites and biocalcarenites up to 300 m thickness. It unconformably overlies the GAKA units and the lower to middle Eocene Ranchuelo Formation. It wedges laterally and south-eastwards into the shallower late Eocene Damují Formation and upwards into the Oligocene Jía Formation (Fig. 1B). Previous biostratigraphical studies were conducted by Bermúdez (1950), who reported smaller late Eocene bathyal benthic assemblages at the type locality. Typical larger late Eocene benthic and planktonic foraminiferal assemblages were reported by García-Delgado & Torres-Silva (1997). Nummulitid specimens from the Jicotea Formation originate from a sample (E-126) collected by Kantshev *et al.* (1976) NW of La Esperanza, Villa Clara Province (22°21′00″N; 80°37′00″W) during geological mapping of central Cuba (Fig. 1A).

#### Arroyo Blanco Formation

The Arroyo Blanco Formation (Hatten *et al*. 1958) forms part of the infill of the Central Basin and extends west and southwards of the Las Villas block, which is structurally related to the La Trocha fault (García-Delgado & Torres-Silva 1997; Cruz-Orosa *et al*. 2012). This unit, between 100 and 150 m thick, is moderately well exposed, especially in the eastern part of Sancti Spiritus and north-east of Sierra de Jatibonico in Sancti Spiritus Province. It is composed of a terrigenous and clastic-carbonate series of polymictic sandstones, conglomerates, calcarenites, biocalcarenites, limestones and marls deposited on a surface of unconformity atop the Remedios Group, the Taguasco Olistostromes and the Vega and Zaza forrmations. The Arroyo Blanco Formation is unconformably overlain by the Jatibonico, Tamarindo and Chambas formations and laterally grades into the Marroquí Formation (García-Delgado & Torres-Silva 1997) (Fig. 1A). The age of the Arroyo Blanco Formation has been attributed as late middle Eocene to late Eocene based on larger benthic and planktonic foraminiferal assemblages (see García-Delgado & Torres-Silva 1997).

Isolated nummulitid specimens were studied at the Loma El Santo section (site label CA-215), representing the lower part of this unit (Fig. 1B). A sequence of about 20 m of hemipelagic marls and argillaceous limestones with interbedded re-sedimented sandstones are exposed 3 km east of Sancti Spiritus, central Cuba (21°55′47″N; 79°26′43.33″W).

#### Blanco Formation

The highest Eocene unit exposed in the Central Basin is the Blanco Formation (Wassall 1955). It was considered to be synonymous with the Upper Oligocene Jatibonico Formation because Wassall (1955) incorrectly identified the Oligo–Miocene species *Lepidocyclina* (*Eulepidina*) *undosa*. This unit has been recently studied by Torres-Silva *et al.* (2017) at Loma Vigía locality (site label CA-216) and attributed a Priabonian age based on the LBF assemblages and calcareous nannofossils. At this locatity, nummulitid specimens were studied from a nearly 60 m thick succession of limestones, marly limestones and marls exposed in a quarry near Siguaney in Sancti Spiritus Province (21°59.483′N; 79°18.680′W) (Fig. 1A).

## Material and methods

### Sample preparation

A total of 112 isolated megalospheric nummulitid specimens were selected and thin-sectioned through the equatorial plane, where the diagnostic internal features are visible and can be measured. Microspheric forms, which are generally rare, were not studied. The investigated specimens originate as follows: Entronque de Herradura (15), Loma Candelaria (39), Loma Jabaco (two), Noroña (15), La Esperanza (9), Loma Vigía (4) and Loma El Santo (14) (see Supplemental material for the distribution of the specimens in the studied sections). Each investigated specimen was photographed and the morphological characters in equatorial sections were measured using the image-processing program ImageJ, version 1.50e. In addition, 14 specimens, including holotypes and/or subsequently published specimens, were measured for taxonomic comparisons.

Eighteen oriented axial sections and more than 100 specimens in petrological sections were studied in order to constrain the stratigraphical and palaeoecological ranges of the nummulitids. The associated LBF assemblages were studied based on 419 oriented individual thin-sections and 43 thin sections of rocks. Planktonic foraminifera and calcareous nannofossils that co-occur with LBF assemblages were studied and assigned to their biozones according to Berggren *et al*. (1995), Martini (1971), Bukry (1973), Pearson *et al*. (2006) and Agnini *et al*. (2014).

Palaeoenvironmental interpretations of the LBF assemblages are broadly based on depositional models for the facies distributions of fossil larger foraminifera (Robinson 1993, 2004; Beavington-Penney & Racey 2004; Ćosović 2004; Bassi 2005). Nummulitid test morphology and variation along the depositional gradient were compared with morphological observations on extant related nummulitid groups (Hohenegger 1999; Yordanova & Hohenegger 2004).

The material is stored at the Department of Palaeontology, University of Vienna, under sample numbers 98LC-1, 98LC-2, LM-52, NOR-UN, 98MT-1, CA-215, CA-216, E-126.

### Morphometry

We present a complete geometric reconstruction of the nummulitid morphology in equatorial sections based on 11 growth-independent and growth-invariant meristic characters (see Hohenegger 2011b; Hohenegger & Torres-Silva 2017). This method enables the classification of the investigated individuals unconstrained by their growth stage, thus improving the interpretation of their systematic and phylogenetic relationships. Measurements of the embryonic apparatus (proloculus, deuteroloculus and first periembryonic chamber) are regarded as growth independent *per se*. Single measurements of chambers recording the character state at a specific position in the spiral were avoided and replaced by a sequence of measurements of one character in every chamber. These sequences can be fitted by growth functions, and parameters of these functions were subsequently used as growth-independent or growth-invariant morphological characters.

**Figure 2.**
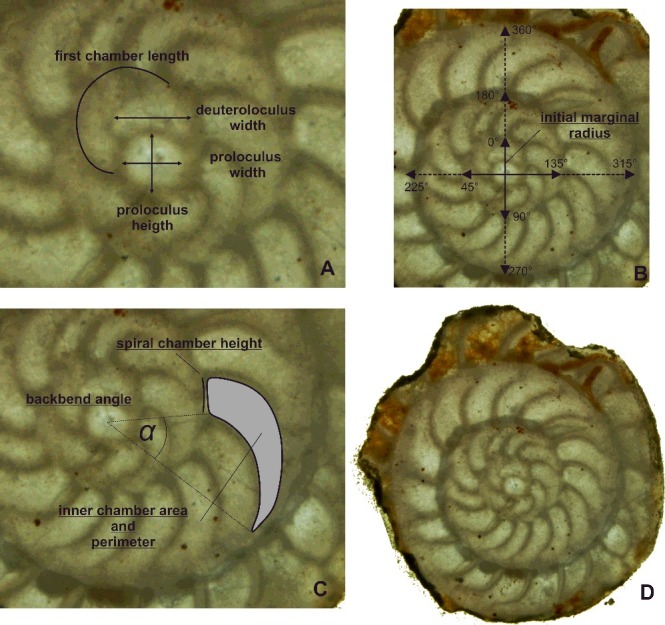
Measurements of characters in equatorial section. **A,** embryonic apparatus; **B,** marginal test spiral; **C,** chamber measurements; **D,** example individual, *Operculinoides floridensis*, specimen 98LC-1H-648.

All characters used in this study either represent or are computed based on measurements of the embryonic apparatus, the marginal spiral and the chamber sequence. Within the embryonic apparatus, proloculus height (**PH**) and width (**PW**), deuteroloculus width (**DW**) and length of the first chamber (**FCL**) were measured (Figure 2). Along the marginal spiral the marginal radius (**MR**) was measured in 45° steps (= 0.785 radians) starting at the initial marginal radius (**IMR**; distance from the centre of the proloculus through the deuteroloculus). The backbend angle (**BBA**), spiral chamber height (**CH**), inner chamber area (**CA**) and inner chamber perimeter (**PER**) were measured for the chamber sequence.

Based on these measurements within the embryonic part, three growth-independent characters can be calculated: proloculus mean diameter (**PD**), deuteroloculus ratio (**DW**) and first chamber length (**FCL**).

The marginal spiral can be described using the marginal radius length (**MR**) as a function of the revolution angle *Ɵ*. The computed parameters of the function initial marginal radius (**IMR**) and marginal radius increase (**MRInc**) represent the characters for the marginal spiral.

Spiral chamber height (**CH**) can be fitted by a linear function, when plotted along the corresponding chamber number. The function parameters initial spiral chamber height (**ICH**) and spiral chamber height increase (**CHInc**) represent growth changes.

For the backbend angle (**BBA**) the arithmetic mean is used as a growth-invariant character, in accordance with Hohenegger & Torres-Silva (2017).

Based on spiral chamber height (CH) and inner chamber area (CA) the theoretical chamber length (**CL**) of every chamber can be calculated using Equation (1):

(1) $$CL_{j}=CA_{j}/CH_{j}$$

*j = chamber number*

The sequential increase of chamber length within the spiral can best be fitted by an exponential function, where the two parameters initial chamber length (**ICL**) and chamber length increase (**CLInc**) represent the change of chamber length through ontogeny. The initial chamber length (ICL) is of similar size to the first chamber length, which is directly measured and influenced by shape and size of the embryonic chambers and is thus omitted from the analysis.

Inner chamber area (CA) and inner chamber perimeter (PER) can further be used to describe the shape of the chambers by relating the perimeter of the chambers to the theoretical perimeter of a square, which is computed using Equation (2):

(2) $$PerR_{j}=Perimeter_{j}/(4\cdot \sqrt{Area_{j}})$$

*j = chamber number*In squared chambers this ratio becomes 1, whereas rectangles with length greater than height yield values > 1, and length smaller than height yields values < 1. Following Hohenegger & Torres-Silva (2017), the arithmetic mean of the perimeter ratio of the chamber (**PerR**) is used as a growth-invariant character.

An overview of all computed characters and how they are obtained is given in Table 1. For a detailed explanation of growth-independent and growth-invariant characters used in these studies, refer to Hohenegger (2011b) and Hohenegger & Torres-Silva (2017).

**Table 1.** Growth-independent and growth-invariant characters.

Character		Attribute	Code	Equation	Growth
Proloculus	1	Proloculus mean diameter	*PD*	$PD\;=\frac{PW+PH}{2}$	Independent
Deuteroloculus	2	Deuteroloculus ratio	*DR*	$DR\;=\frac{DW}{PW}$	Independent
First chamber	3	First chamber length	*FCL*	Directly measured	Independent
Marginal test spiral	4	Initial radius	*IMR*	$MR\;=\;IMR\cdot e^{MRInc\;\;\theta }$	Independent
*RL*	5	Radius increase	*MRinc*		Invariant
Spiral chamber height	6	Initial spiral chamber height	*ICH*	CH = CHinc⋅j+ICHex	Independent
*CH*	7	Spiral chamber height increase	*CHinc*		Invariant
Backbend angle *BBA*	8	Backbend angle	*BBA*	$BBA\;=(1/n\;)\sum_{j\;=\;1}^{j\;=\;n}{BBA}_{j}$	Invariant
Chamber lengths	9	Initial final chamber length	*ICL*	$CL\;=\;ICL\cdot e^{CLinc}$	Independent
	10	Final chamber length increase	*CLinc*		Invariant
Perimeter ratio *PerR*	11	Perimeter ratio of chambers	*PerR*	$PerR\;=(1/n\;)\sum_{j\;=\;1}^{j\;=\;n}{PerR}_{j}$	Invariant

Since investigated growth-invariant and growth-independent characters (**k**) have different scales in the case of direct measurements or are dimensionless, but with different range widths, they have been standardized to normal distributions (x*) with a mean = 0 and standard deviation = 1.

### Data processing

The 112 specimens were classified based on the standardized values of all investigated growth-independent and growth-invariant characters using an agglomerative cluster analysis (K-means algorithm; Bow 1984). A principal component analysis (PCA) based on standardized Euclidean distances was used to represent specimens in a two- or three-dimensional space to detect the concentration of individuals separated by gaps from other concentration centres. Supported by the results of the cluster analyses, all specimens were separated into clusters, which can be later interpreted as genera, species, ecomorphotypes or stratigraphically separated groups. The importance of characters for this separation between and within morphospecies was checked by canonical discriminant analyses (CDA). Differences between the proposed genera were additionally tested for every character by analysis of variance (ANOVA). Furthermore, differences between species were also tested for every character and attribute by ANOVA. Both analyses are followed by a Tamhane T2 post-hoc multiple comparison test to detect significant differences for each character between groups.

Subsequently, a CDA was run within each species to check for stratigraphical or palaeoecological differences in morphology between the studied localities. Finally, five specimens of *Heterostegina ocalana*, five of *H. cubana* and two of *H.* sp. indet. (Torres-Silva *et al*. 2017) were included in a PCA to check for potential morphological relationships. The growth-independent and growth-invariant characters used in Hohenegger & Torres-Silva (2017) were transformed and reduced to gain the same set of characters investigated in all nummulitids. The set of characters used in this investigation emphasizes the relationship of *Heterostegina* to the studied nummulitds rather than the relationship between different *Heterostegina* species, because morphological characters describing chamberlets were disregarded in the present study. The importance of characters has been additionally supported by discriminant analysis.

Discriminant analysis and ANOVA including post-hoc tests were done using IBM SPSS Statistics 22, and for cluster and ordination analysis PAST 3.02 was used, whereas simpler calculations were performed in Microsoft Excel 2013.

## Results

### Statistical results

**Figure 3.**
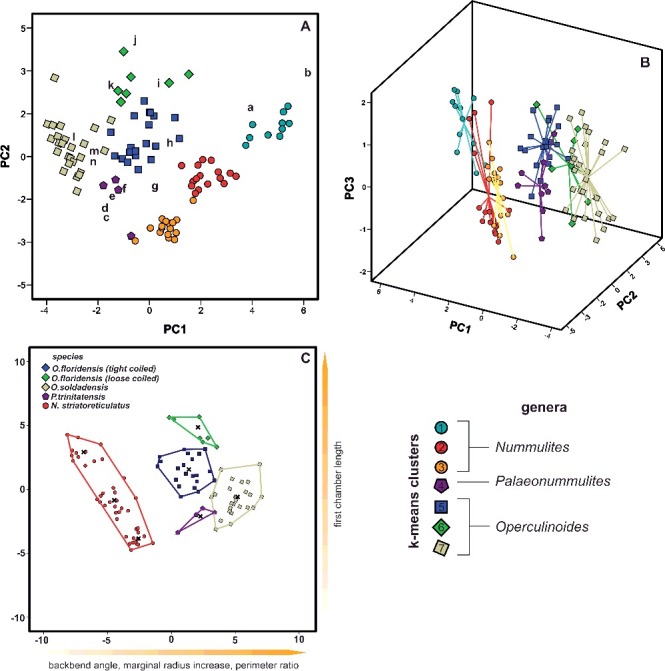
**A,** two-dimensional ordination of studied specimens; colours accord with the results of the K-means cluster analysis. Numbers indicate the measured type material: 1, *Nummulites stritoreticulatus*, holotype; 2, *N. macgillavry* (from Butterlin 1981); 3, *Operculinoides trinitatensis*, holotype; 4, *O. spiralis*, holotype; 5. *O. kugleri*, holotype; 6, *N. trinitatensis* (from Butterlin 1961); 7, *O. willcoxi* (from Barker 1939); 8, *O. willcoxi* (from Cole 1941); 9, *O. floridensis* (from Frost & Langenheim 1974); 10, *O. floridensis* (from Cole 1941); 11, *O. floridensis* (from Cole 1941); 12, *O. soldadensis* (from Vaughan & Cole 1941); 13, *O. suteri* (from Caudri 1996); 14, *N. floridensis* (from Butterlin 1961). **B,** three-dimensional ordination of the studied specimens emphasizes the variation in the third component, highlighting the differentiation between *Nummulites* from 98LC-2 and *Palaeonummulites* from 98LC-1. **C,** discriminant analysis between the interpreted species: *Nummulites striatoreticulatus*, *Palaeonummulites trinitatensis*, *Operculinoides floridensis* and *Operculinoides soldadensis*; parameters are sorted in order of their importance as discriminators.

The classification of 112 specimens using six growth-independent and five growth-invariant characters yielded seven distinct clusters using K-means clustering and PCA. The PCA has been illustrated in a two-dimensional space (Fig. 3A), where polygons highlight the resulted clusters, as well as in a three-dimensional space (Fig. 3B). This emphasizes the difference between overlapping clusters in the third component (Fig. 3B). The results of the k-means analysis are illustrated in the matching ordination (Fig. 3C).

Cluster 1 includes the holotype of *Nummulites striatoreticulatus* Rutten, 1928 from Curaçao and one Mexican specimen of *N. macgillavry* (Butterlin, 1981). Cole (1958) only reported two *Nummulites* sensu stricto species from the American-Caribbean province. Due to the close proximity of the 43 Cuban specimens to the *N. striatoreticulatus* holotype, we regard clusters 1, 2 and 3 as *Nummulites* sensu stricto. The specimen of *N. macgillavry* plots farthest away from the centroid of cluster 1 and is hence regarded as not present in the studied locations. Even though clusters 1–3 show a morphological differentiation, all groups are regarded as *N. striatoreticulatus* because they plot relatively close to the type of *N. striatoreticulatus* and show characters of true *Nummulites*. Four Cuban specimens of cluster 4 plot together with the type material of *Operculinoides trinitatensis*, *O. spiralis* and *O. kugleri* from Trinidad, as well as with *O. willcoxi* (Barker, 1939). The whole cluster is regarded as the genus *Palaeonummulites* and the species *P. trinitatensis* (see Systematic palaeontology).

Cluster 5 includes the holotypes of *O. soldadensis* and *O. suteri* from Trinidad, cluster 6 *O. willcoxi* (Cole, 1941) from Florida, and cluster 7 two specimens of *O. floridensis* from Florida (Cole 1941) and one from Mexico (Frost & Langenheim 1974). Hence, the 52 Cuban specimens belonging to these three clusters are denoted as *Operculinoides* Hanzawa, 1935 (see Systematic palaeontology). According to the presence of holotypes in cluster 5, it is regarded as *O. soldadensis* (which is synonymous with *O. suteri*), cluster 6 is regarded as a tightly coiled morphotype of *O. floridensis*, and cluster 7 is regarded as a loosely coiled morphotype of *O. floridensis.* One specimen can be regarded as *O. ocalanu*s (CA4-724) but was not included in the analysis.

Significant differences between genera (*Nummulites*, *Palaeonummulites* and *Operculinoides*) have been revealed in each character except for first chamber length (FCL). This is further specified by a post-hoc multiple comparison (see Systematic palaeontology). Likewise, significant differences between the assigned species (*N. striatoreticulatus*, *P. trinitatensis*, *O. soldadensis*, *O. floridensis*) have been highlighted by ANOVA analysis and post-hoc multiple comparison. The importance of characters for the differentiation of morphospecies has been additionally underlined by CDA (Fig 3C). Discriminant functions 1 and 2 explain 89.0% of variance between the species. They differ along function 1 in the backbend angle (BBA), marginal radius (MR) and perimeter ratio (PerP), and along function 2 in first chamber length (FCL). This analysis, however, mainly emphasizes the differences between *N. striatoreticulatus* and the operculinid species.

**Figure 4.**
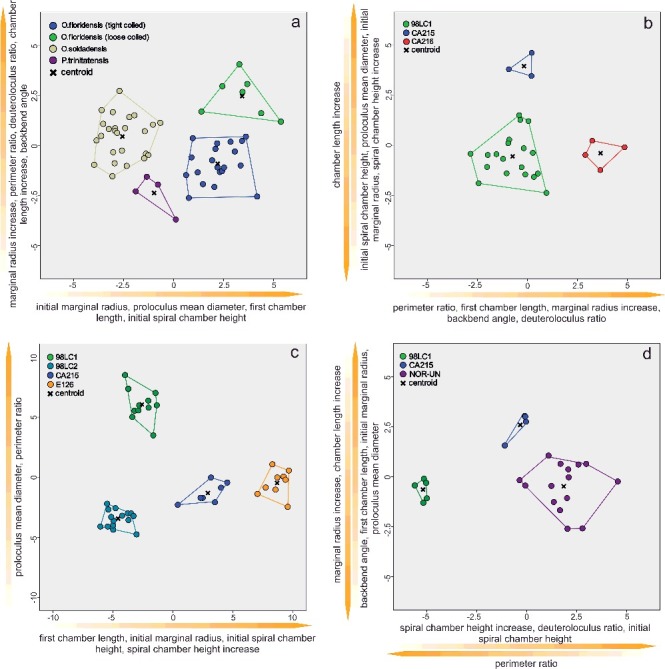
Discriminant analysis of nummulitid species, where the important discriminators are ranked along the discriminant functions. **A,** discriminant analysis between *Operculinoides* and *Palaeonummulites* species; **B,** discriminant analysis within *Nummulites striatoreticulatus* from different localities; **C,** discriminant analysis within *O. floridensis* from different localities; **D,** discriminant analysis within *O. soldadensis* from different localities.

Therefore, a CDA was performed to evaluate differences in morphological characters between species of *Palaeonummulites* and *Operculinoides*. Discriminant functions 1 and 2 explain 95.3% of the variance between the *Operculinoides* species (Fig. 4A). *Operculinoides soldadensis* differs from *O. floridensis* (tightly coiled) mostly along function 1 (abscissa), namely by a smaller initial marginal radius (IMR), proloculus diameter (PD), first chamber length (FCL) and initial spiral chamber height (ICH), and slightly along function 2 in a stronger marginal radius increase (MRInc), perimeter ratio (PerP), deuteroloculus ratio (DW), chamber length increase (CLInc) and backbend angle (BBA). *Operculinoides floridensis* (tightly coiled) differs from *O. floridensis* (loosely coiled) mostly along function 2 (ordinate), namely by a stronger marginal radius increase (MRInc), perimeter ratio (PerP), deuteroloculus ratio (DW), chamber length increase (CLInc) and backbend angle (BBA). *Operculinoides floridensis* (loosely coiled) differs from *O. soldadensis* along function 1 (abscissa) by a smaller initial marginal radius (IMR), proloculus diameter (PD), first chamber length (FCL) and initial spiral chamber height (CH), and along function 2 (ordinate) by a stronger marginal radius increase (MRInc), perimeter ratio (PerP), deuteroloculus ratio (DW), chamber length increase (CLInc) and backbend angle (BBA).

CDA within the species revealed morphological changes between specimens from different localities. Due to the low sample size of cluster 6 and the morphological similarity of clusters 5 and 6, tightly and loosely coiled specimens of *O. floridensis* were grouped together to check for intraspecific differences between localities.

In the *O. floridensis* group, discriminant functions 1 and 2 explain 100% of variance (Fig. 4B), where main differences between localities 98LC-1 and CA-216 are expressed along the abscissa in perimeter ratio (PerP), first chamber length (FCL), marginal radius increase (MRInc), backbend angle (BBA) and deuteroloculus ratio (DW), and differences between localities 98LC-1 and CA-215 are expressed along the ordinate in larger initial spiral chamber height (CH), proloculus diameter (PD), initial marginal radius (IMR), spiral chamber height increase (CHInc) and chamber length increase (CLInc).

In *O. soldadensis*, discriminant functions 1 and 2 explain 100% of variance (Fig. 4D), where main differences between localities 98LC-1 and NOR-UN are expressed along function 1 (abscissa) only in smaller spiral chamber height increase (CHInc), smaller deuteroloculus ratio (DW), smaller initial spiral chamber height (CH) and larger perimeter ratio (PerP). Differences between localities CA-215 and 98LC1 are expressed along function 1 in smaller spiral chamber height increase (CHInc), smaller deuteroloculus ratio (DW), smaller initial spiral chamber height (CH) and larger perimeter ratio (PerP), and along function 2 in a larger marginal radius increase (MRInc), larger chamber length increase (CLInc), smaller backbend angle (BBA), smaller first chamber length (FCL), smaller initial marginal radius (IMR) and smaller proloculus nominal diameter (PD).

**Figure 5.**
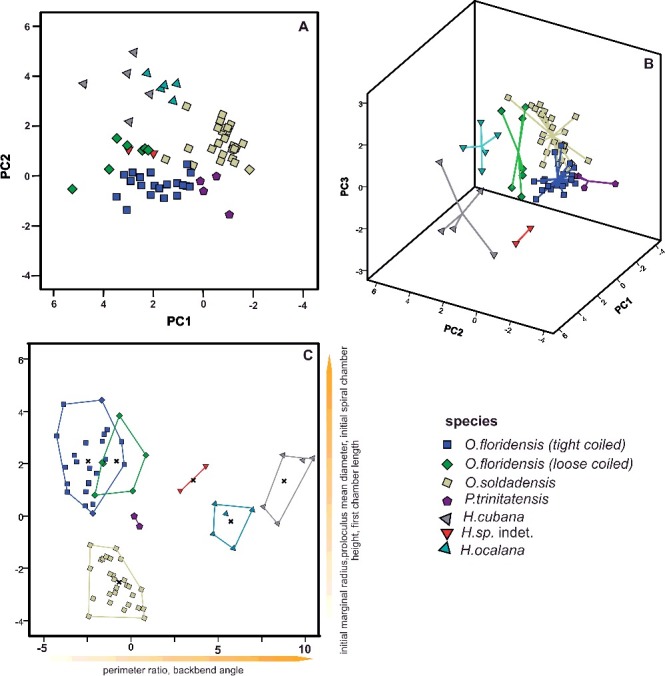
Ordinations and discriminant analysis. **A,** two-dimensional ordination of studied specimens; genera are separated by different shapes (squares = *Operculinoides*; polygons = *Palaeonummulites*; triangles = *Heterostegina*). **B,** three-dimensional ordination of the studied specimens emphasizes the variation in the third component, highlighting the differentiation between *Heterostegina* sp. indet. *and Operculinoides.* **C,** discriminant analysis of *Heterostegina* species and *Operculinoides* or *Palaeonummulites* species; parameters are sorted in order of their importance as discriminators.

Within *N. striatoreticulatus*, discriminant functions 1 and 2 explain 97.8% of the variance between different localities (Fig. 4C). Specimens from 98LC-2 differ from those of 98LC-1H in a smaller proloculus mean diameter (PD) and perimeter ratio (PerR). 98LC-2 in comparison to specimens from CA-215 and E-126 differs less in these three characters, but does differ in first chamber length (ICL), initial marginal radius (IMR) and initial spiral chamber height (ICH), as well as in a higher spiral chamber height increase (CHInc). Figure 5A and B illustrates the morphological distance of *H. ocalana*, *H. cubana* and *H*. sp. indet. to the different operculinid species in two- and three-dimensional space, where *H.* sp. indet. is positioned nearest to *O. floridensis*. A further canonical discriminant analysis, where functions 1 and 2 explain 97.7% of the variance, revealed the importance of characters for this morphological relationship. Along the first function, *H*. sp. indet. is positioned between *O. floridensis* and the other two *Heterostegina* species due to perimeter ratio (PerR) and backbend angle (BBA). In function 2, its values in initial marginal radius (IMR), proloculus nominal diameter (PD), initial spiral chamber height (ICL) and first chamber length (FCL) position it nearer to *O. floridensis* than *P. trinitatensis*.

Additional information on the PCA, CDA, ANOVA and post-hoc multiple comparisons is given in the Supplemental material.

### Biostratigraphy and palaeoenviromental inferences

Fundamental for the biostratigraphical and palaeoecological results presented herein are the Caribbean occurrences of orbitoids and nummulitids, including *Lepidocyclina*, *Helicostegina*, *Eulinderina*, *Discocyclina*, *Asterocyclina*, *Pseudophragmina*, *Heterostegina Nummulites*, *Palaeonummulites* and *Operculinoides*, and most of the Palaeogene amphisteginids and agglutinated conical forms (Cole 1958; Butterlin 1981; Robinson & Wright 1993; Caudri 1996; Torres-Silva *et al*. 2017). These assemblages are geographically widespread in the American- Caribbean province and were deposited in a variety of settings on the outer margins of the carbonate platforms. Robinson (1993, 2004) recognized these as Assemblage I, and because of their typical (palaeo)habitats at the edge of shallow water areas, they were susceptible to post-mortem downslope displacement. Thus, they are frequently found as penecontemporaneous re-sedimented components of turbidites in hemipelagic sequences related to arc/subduction tectonic situations in the Caribbean, e.g. Cuba (Brönnimann & Rigassi 1963; García-Delgado & Torres-Silva 1997; Molina *et al*. 2016; Torres-Silva *et al*. 2017), Trinidad (Vaughan & Cole 1941), Venezuela (Caudri 1974) and Jamaica (Robinson 1993). Assemblage II, restricted mainly to the back reef and interior shelf environments and characterized by imperforate assemblages including *Yaberinella*, *Fabularia*, *Colecoinus*, *Pseudochrysalidina*, *Verseyella* and *Peneroplis*, was not found at the Cuban localities and appears to be endemic to the Nicaragua Rise (Robinson 2004).

**Figure 6.**
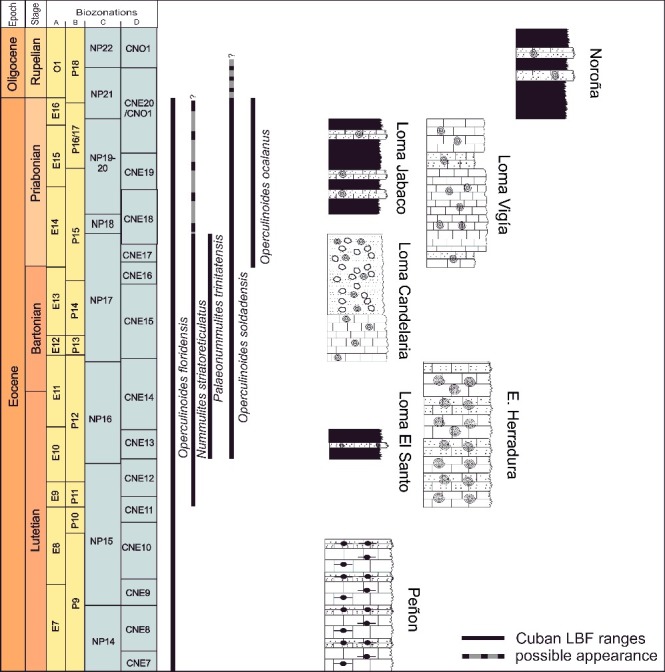
Stratigraphical ranges of the nummulitid species from the Cuban sections and their correlation with the standard planktonic zones. **A,** Pearson *et al.* (2006); **B,** Berggren *et al.* (1995); **C,** Martini (1971); **D,** Agnini *et al.* (2014).

**Figure 7.**
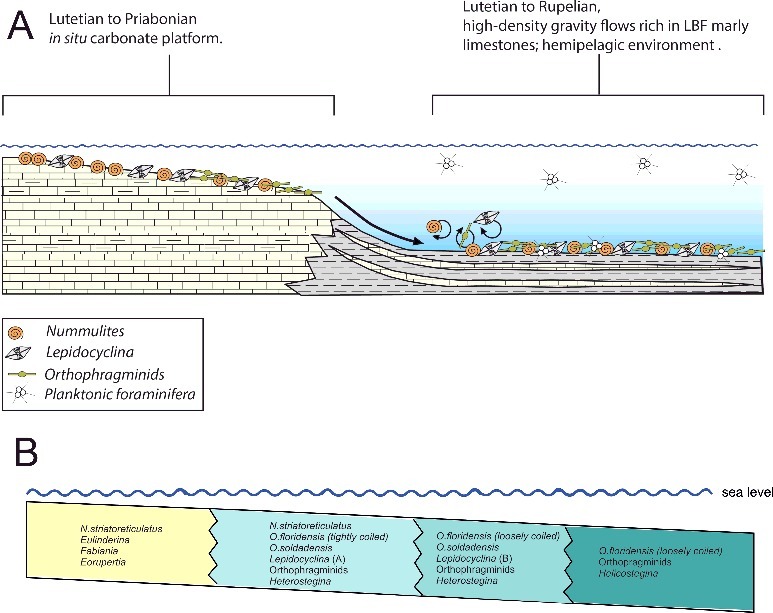
**A,** schematic diagram showing the Eocene depositional environments in sections from western and central Cuba (modified from Cotton 2012). **B,** schematic diagram showing depth zonation of nummulitid species and larger benthic foraminifera (LBF) present in the Eocene section across the depositional gradient (modified from Beavington-Penney & Racey 2004).

**Figure 8.**
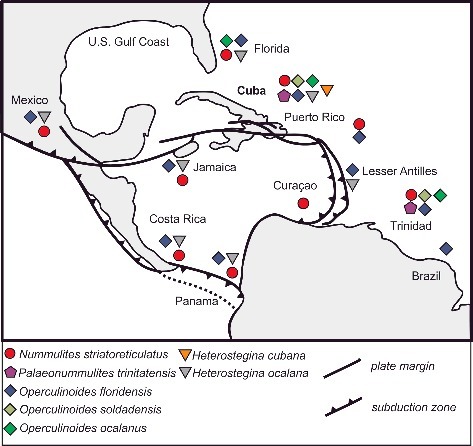
Palaeogeographical distribution of the Eocene nummulitid species found at the Cuban localities. Map adapted from Pindell (2009).

Biostratigraphical and palaeoecological inferences of the investigated localities are presented below. Stratigraphical ranges, approximate palaeodepth ranges and palaeobiogeographical distributions of the nummulitid species recognized herein are summarized in Figures 6–8.

**Figure 9.**
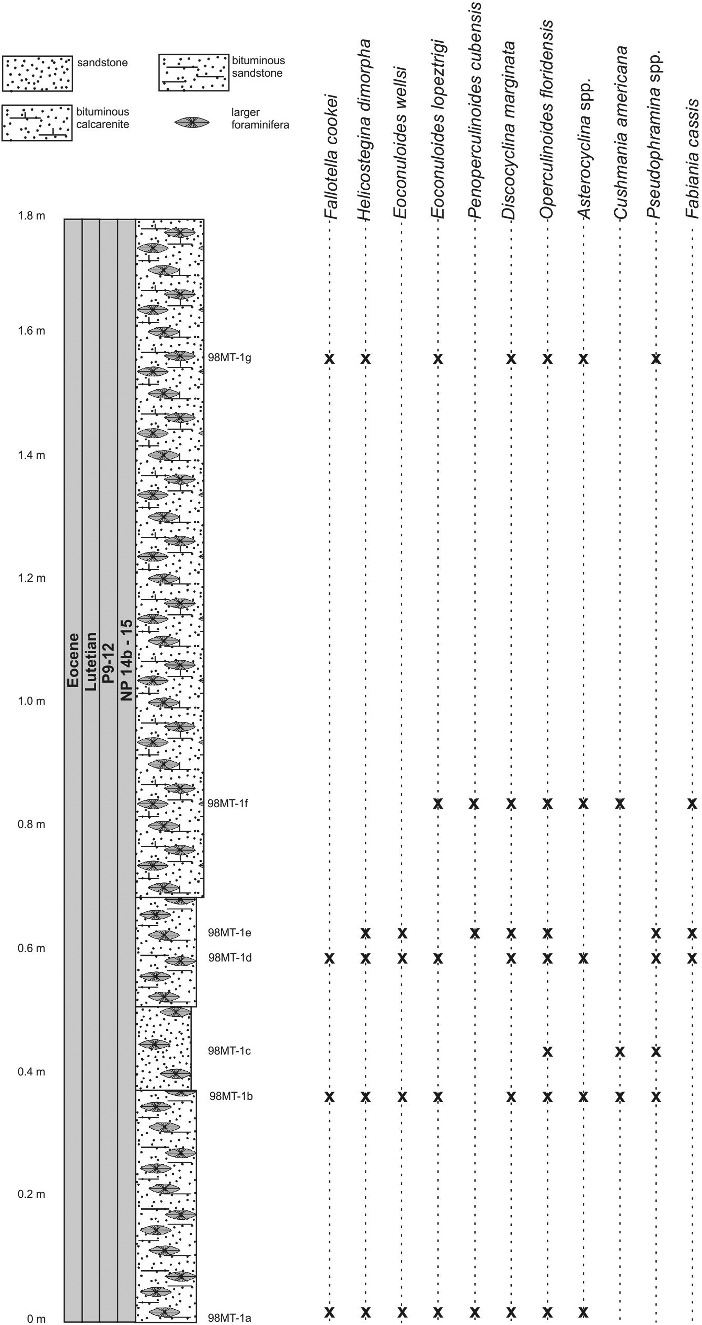
Distribution of larger benthic foraminifera (LBF) in the Angelita Quarry section, western Cuba.

At Angelita Quarry (98MT-1) the LBF are fairly consistent throughout the section and typical for the Caribbean earliest middle Eocene consisting of *Helicostegina dimorpha*, *Eoconuloides wellsi*, *E. lopeztrigoi*, *Gunteria floridana*, *Discocyclina marginata*, *Cushmania americana*, *Fallotella cookei*, *Fabiania cassis*, *Operculinoides floridensis* and several species of *Asterocyclina*, *Discocyclina* and *Pseudophragmina* (Fig. 9). The overlap of *Operculinoides floridensis* with *Helicostegina dimorpha* exemplifies the first stratigraphical appearance of nummulitids in the studied localities (Fig. 6). Robinson (2004) correlated the first appearance of *Helicostegina dimorpha* and *Nummulites* sensu lato in the White Limestone Group of Jamaica with the *Helicostegina-Nummulites* subzone corresponding to the early middle Eocene of calcareous nannofossil zones NP 14b and NP 15. The dominance and high diversity of the usually very thin orthophragminids and the occurrences of loosely coiled forms of *Operculinoides floridensis* at this locality indicate open marine, outer shelf conditions corresponding to the deeper part of the photic zone (Fig. 7). According to Ćosović *et al*. (2004), the diversity of orthophragminids increases with progressive depth of deposition. Test flattening and wall thinning in extant nummulitids is influenced by light intensity and water energy, both factors negatively correlated with water depth increase (e.g. Hohenegger 1999; Beavington-Penney & Racey 2004; Yordanova & Hohenegger 2004).

**Figure 10.**
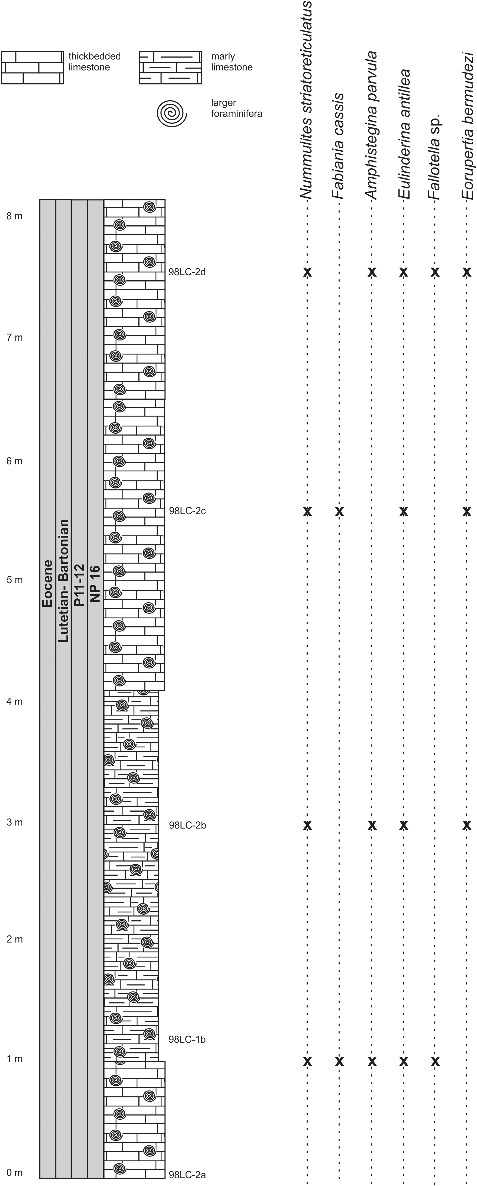
Distribution of larger benthic foraminifera (LBF) in the Entronque de Herradura section, western Cuba.

**Figure 11.**
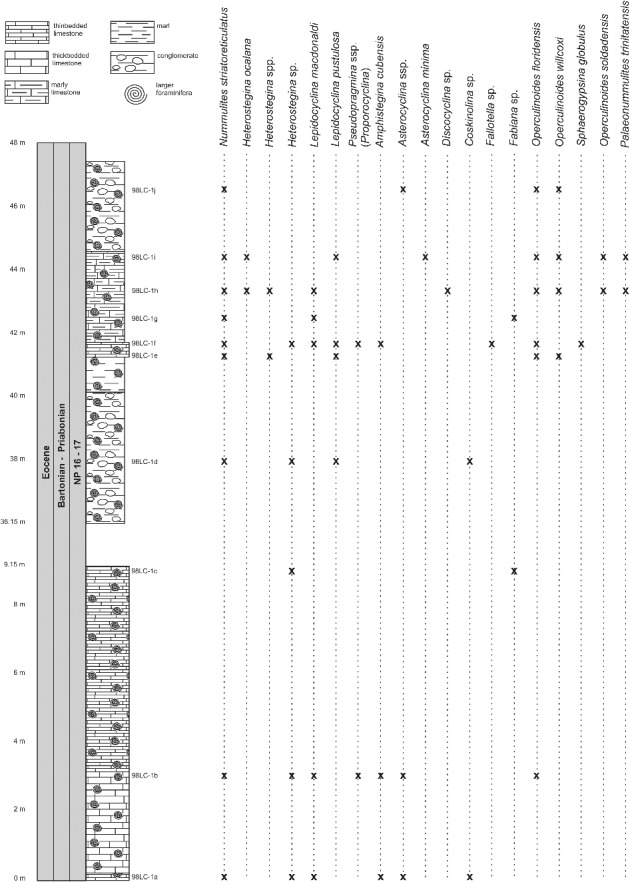
Distribution of larger benthic foraminifera (LBF) in the Loma Candelaria section, western Cuba (modified from Torres-Silva *et al*. 2017).

The lower part of the Loma Candela Formation (98LC-2) is dominated by robust and lenticular specimens of *Nummulites striatoreticulatus* associated with *Eulinderina antillea*, *Fabiania cassis*, *Amphistegina parvula*, *Eorupertia bermudezi* and *Fallotela floridana* (Fig. 10). Robinson (1996, 2004) reported the occurrence in the Gulf of Mexico and the northern Caribbean of *Eulinderina antillea* through planktonic foram zones P11 to P12 (Berggren *et al*. 1995) and calcareous nannofossil zone NP 16 (Martini 1971). Based on these results we correlate this section and the first appearance of *Nummulites striatoreticulatus* to the early middle Eocene (Lutetian) (Fig. 6). *Nummulites* sensu stricto, like *Nummulites striatoreticulatus*, is a characteristic inhabitant of shelf edge environments in the Caribbean province (Robinson 2004) (Fig. 8). The absence of lepidocyclinids, orthophragminids and operculinoids at locality 98LC-2 is evidence for the shallowest environmental conditions of the studied localities (Fig. 7). A slight increase in water depth is registered in the upper part of the Loma Candela Formation (98LC-1), with the overlap of *Nummulites striatoreticulatus* with less abundant, tightly to loosely coiled forms of *Operculinoides floridensis* and *O. soldadensis* and its association with abundant megalospheric lepidocyclinids, less abundant orthophragminids and heterosteginids (Figs 7, 11).

**Figure 12.**
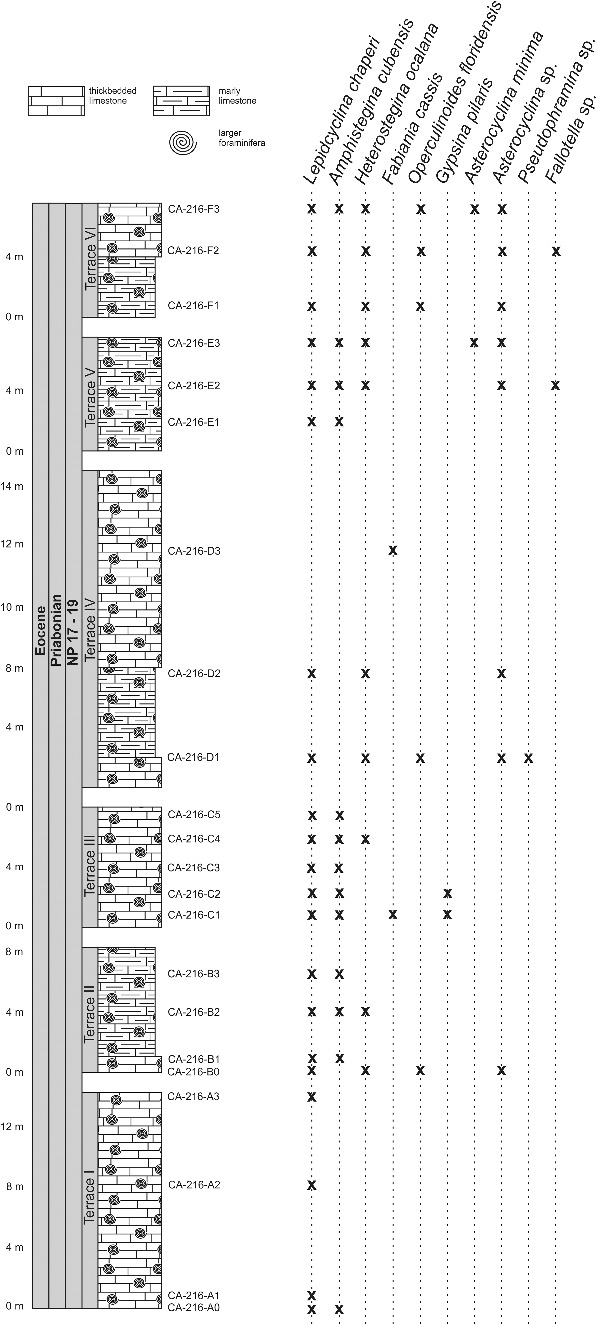
Distribution of larger benthic foraminifera (LBF) in the Loma El Santo section, central Cuba.

At the Loma El Santo section (CA-215), corresponding to the lower part of the Arroyo Blanco Formation, *Nummulites striatoreticulatus*, *Operculinoides floridensis* and *O. soldadensis* were found abundantly in sandy samples re-sedimented from the shelf and interpreted to have been deposited penecontemporaneously with the surrounding marls and argillaceous limestones. These lithologies contain abundant planktic foraminifera and calcareous nannofossils. The most characteristic planktic foraminiferal species are *Hantkenina alabamensis*, *Morozovella aragonensis*, *Globigerinatheka mexicana*, *Igorina brodermani*, *Pseudohastigerina micra* and *Acarinina* sp. Following Pearson *et al*. (2006), zones E10 and E11 (Lutetian, middle Eocene) were identified. These biozones correlate with the P12 zone (Berggren *et al*. 1995). The calcareous nannofossils are highly diverse and very well preserved, and the most characteristic species are *Sphenolithus furcatolithoides*, *S. cuniculus* and *Reticulofenestra umbilicus.* Stratigraphical attribution to NP16 Zone (Martini 1971) is based on the absence of *Blackites gladius* (Locker, 1967) (Varol 1989) and the presence of *R. umbilicus*. More precise biostratigraphical attribution allows the zonation defined by Agnini *et al*. (2014). Investigated samples can be attributed to Zone CNE13 (*Reticulofenestra umbilicus* Base Zone) with an estimated age of 43.06–42.37 Ma, corresponding to middle to late Lutetian. The penecontemporanous re-deposition of the LBF into the hemipelagic palaeoenviroment points to a middle to late Lutetian age (Fig. 12). The overlapping niches of *Nummulites striatoreticulatus* with the tightly to moderately coiled *Operculinoides* species and their association with *Lepidocyclina macdonaldi*, *Cushmania americana*, *Amphistegina parvula* and *Asterocyclina havanensis* suggest a carbonate source at the shelf edge or shallowest parts of the upper slope (Fig. 7).

Occurrences of the largest and flattest *Nummulites striatoreticulatus* individuals were found at sample site E-126 in the Jicotea Formation, associated with *Lepidocyclina pustulosa*, *L. chaperi* and *Pseudophragmina* sp., and probably represent the deepest *N. striatoreticulatus* populations in the Cuban localities. The LBF association can be attributed to the Priabonian (late Eocene).

**Figure 13.**
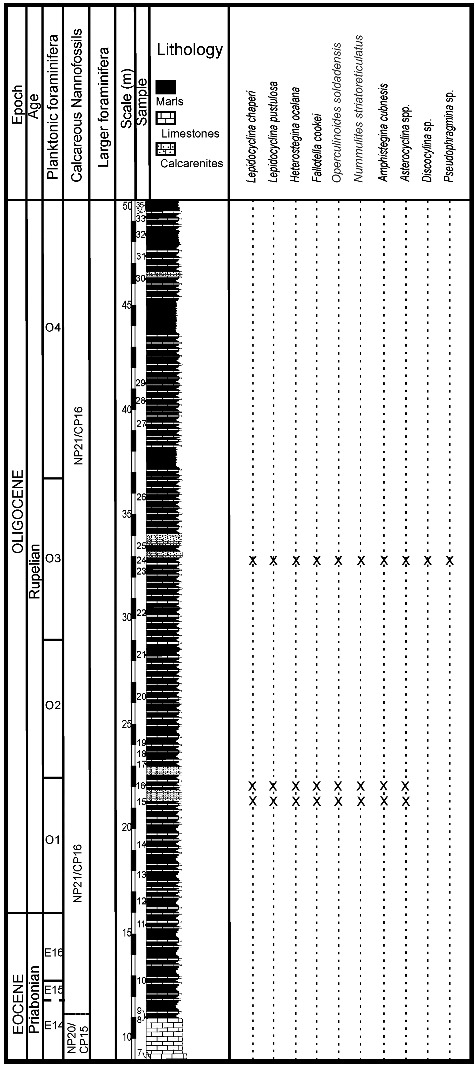
Distribution of larger benthic foraminifera (LBF) in the Noroña section, western Cuba (modified from Torres-Silva *et al.* 2017).

**Figure 14.**
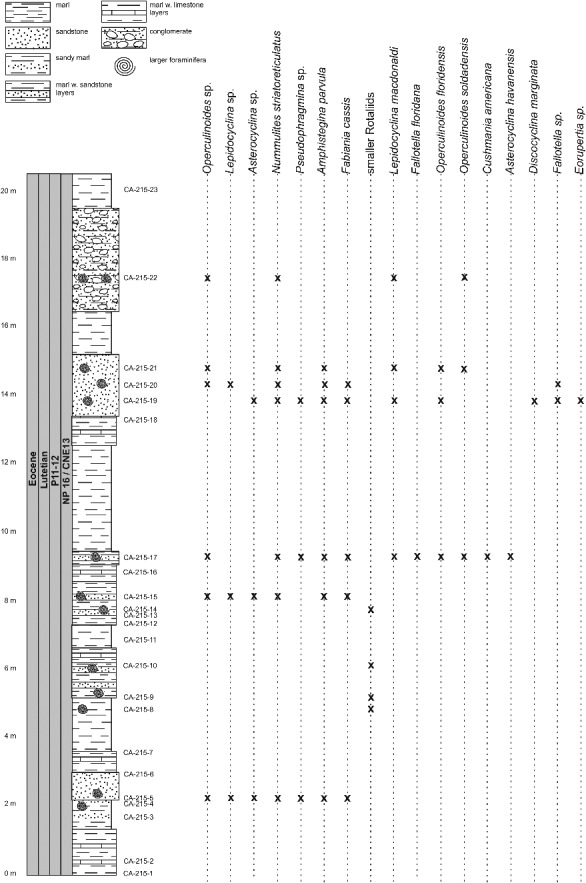
Distribution of larger benthic foraminifera (LBF) in the Loma Vigía section, central Cuba.

At Norona section (NOR-UN), of possible Rupelian age (Fig. 13), the extremely rare presence of *Nummulites striatoreticultus* (two specimens) and the predominance of *Operculinoides soldadensis* and *Heterostegina ocalana* together with megalospheric *Lepidocyclina pustulosa* suggest a source in a distal part of the upper slope (Fig. 7). Deeper, open marine shelf conditions can be deduced at Loma Vigía section (CA-216), supported by the absence of *Nummulites striatoreticulatus* and the abundances of loosely coiled forms of *Operculinoides floridensis* and *Heterostegina ocalana* associated with enormous numbers of microspheres of the species *L. chaperi* (Figs 7, 14). This is in accordance with the assumption that sexual reproduction is likely restricted to deeper environments below fair-weather wave base (Beavington-Penney & Racey 2004; Eder *et al*. 2017a).

## Systematic palaeontology

### 

#### Remarks

The analysis based on the six growth-independent and five growth-invariant characters resulted in a classification of the Eocene nummulitids into four species in three genera: *Nummulites*, *Palaeonummulites* and *Operculinoides* (Fig. 4). Qualitative characters, such as the relative amount of involution, development of the marginal cord and character of the septa, were also considered important for differentiation at the generic level. The presence or absence of trabeculae and the type of stolon system as proposed by Hottinger (1997) as important characters at the generic level proved to be of limited use in the fossil record because they are visible only on exceptionally well-preserved specimens.

In addition to the differential diagnoses of the species here described, tables are presented for each species containing character means and standard deviations combined with statistically significant differences between related species.

Order **Foraminiferida** Eichwald, 1830

Suborder **Rotaliina** Delage & Hérouard, 1896

Superfamily **Nummulitoidea** de Blainville, 1827

Family **Nummulitidae** de Blainville, 1827

Subfamily **Nummulitinae** de Blainville, 1827

Genus ***Nummulites*** Lamarck, 1801

#### Type species

*Nummulites laevigatus* (Bruguiére, 1792).

#### Diagnosis

Planispiral, involute, lenticular to globular, spire tight with numerous whorls. Many simple chambers per whorl, which are rather equidimensional, septa curved back at the periphery and may be sigmoidal. The marginal cord is well developed.

Characters and attributes (means and standard deviations) for *Nummulites* and comparison with *Palaeonummulites* and *Operculinoides* are given in Table 2.

**Table 2.** Characters and attributes (means and standard deviations, SD, in mm) for *Nummulites* and comparisons with *Palaeonummulites* and *Operculinoides.* Symbol key: ++, strong positive differences with < 1% error probability; +, differences with < 5% error probability; 0, no significant differences; −−, strong negative differences with < 1% error probability.

*Nummulites*	Mean	SD	*Operculinoides*	*Palaeonummulites*
First chamber length	448.7	220.50	0	++
Proloculus nominal diameter	334.2	152.84	++	++
Deuteroloculus ratio	0.999	0.1297	−−	0
Initial marginal radius	355.9	143.51	++	++
Marginal radius increase	0.062	0.0081	−−	0
Spiral chamber height increase	3.8	1.47	++	++
Initial spiral chamber height	111.0	58.49	++	++
Backbend angle	0.164	0.0513	−−	+
Initial chamber length	246.6	125.21	0	++
Chamber length increase	0.009	0.0065	−−	0
Perimeter ratio	1.101	0.0595	−−	0

#### Range

Late Paleocene to Oligocene.

***Nummulites striatoreticulatus*** Rutten, 1928

(Fig. 15A–M)

**Figure 15.**
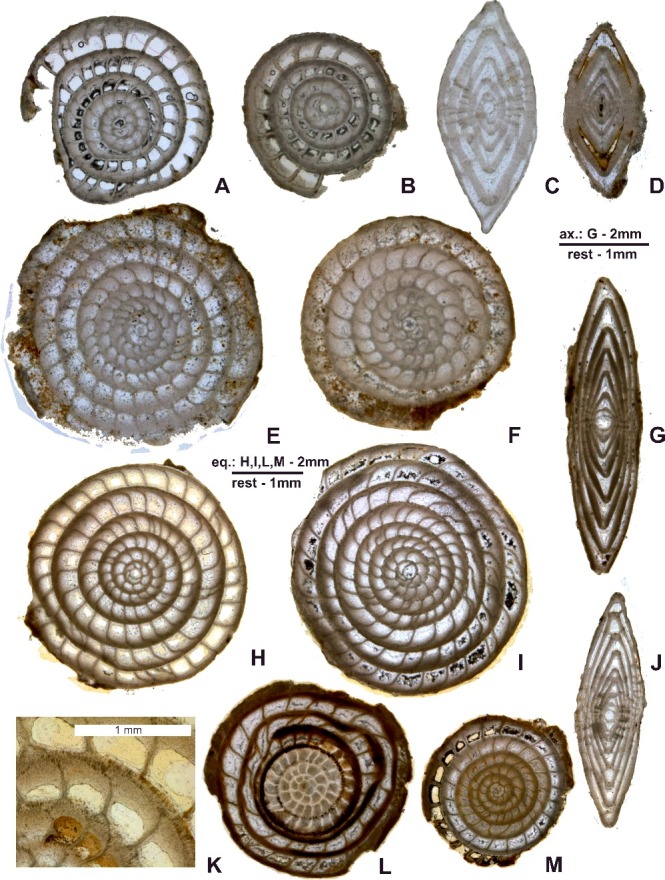
*Nummulites striatoreticulatus* Rutten. **A–C,** Entronque de Herradura; **A,** 98LC-2-686; **B,** 98LC-2-687; **C,** 98LC-2-1a. **D–F,** Loma Candelaria; **D,** 98LC-1-660; **E,** 98LC-1-630; **F,** 98LC-1-806. **G–K,** La Esperanza; **G,** E-126-474; **H,** E-126-466; **I,** E-126-458; **J,** E-126-470, gaps in the septa between adjacent alar prolongations of the chambers; **K,** E-126-459; **L, M,** Loma El Santo; **L,** CA-215- 865; **M,** CA-215- 65. **A, B, E, F, H, I, L** and **M** are A forms in equatorial section; **C, D, G** and **J** are A forms in axial section.

1928 *Nummulites striatoreticulatus* Rutten: 1068, pl. 1, figs 41–50, F–J.

1941 *Camerina vanderstoki* (Rutten & Vermunt); Cole: 28, pl. 8, figs 5, 8.

1942 *Camerina vanderstoki* (Rutten & Vermunt); Cole: 27, pl. 8, fig. 10.

1958 *Camerina striatoreticulata* (Rutten); Cole: 265, pl. 32, figs 6–8.

1974 *Nummulites* (*Nummulites*) *striatoreticulatus* Rutten; Frost & Langenheim: 74, pl. 11, figs 1–14, pl. 13, figs 1, 13.

1993 *Nummulites striatoreticulatus* Rutten; Robinson & Wright: 331, pl. 30, fig. 5, pl. 30, fig. 6.

#### Material

Sixty-one well-preserved megalospheric specimens comprising 15 equatorial sections from Entronque de Herradura (98LC-2); 12 equatorial and nine axial sections from Loma Candelaria (98LC-1); seven equatorial sections from Loma El Santo (CA-215); and nine equatorial and nine axial sections from (E-126).

#### Description

 

##### External features

The test is planispiral involute, inflated, biconvex with a lenticular contour and a diameter in the A form ranging from 1.7 to 8.5 mm. Surface smooth with radial septal traces forming distinctly raised lines radiating from the centre to the periphery.

##### Internal features

The embryonic apparatus is bilocular, proportionally small for the test size. Subspherical proloculus ranging from 0.12 to 0.40 mm followed by a reniform deuteroconch about 0.10 to 0.56 mm in diameter. Spiral exhibits a weaker marginal radius increase, producing numerous whorls. There are many simple chambers that are more or less equidimensional in the equatorial plane. In some specimens, chambers in the outer whorls can be up to 2 times as long as high. Chambers are divided by septa gently bent inwards (weak backbend angle), and supplementary passages can be present as a result of gaps in the septa between adjacent alar prolongations of the chambers. The well-developed marginal cord, with a fan-shaped cluster of coarse canals, forms the chamber apex. Pillars visible in axial section usually do not reach the surface of the test.

Characters and attributes (means and standard deviations) for *Nummulites striatoreticulatus* and comparison to *Palaeonummulites trinitatensis*, *Operculinoides floridensis* (tightly coiled) and *O. floridensis* are given in Table 3.

**Table 3.** Characters and attributes (means and standard deviations, SD, in mm) for *Nummulites striatoreticulatus* and comparisons with *Palaeonummulites trinitatensis*, *Operculinoides floridensis* (tightly coiled), *O. floridensis* (loosely coiled) and *O. soldadensis.* Symbol key: ++, strong positive differences with < 1% error probability; +, differences with < 5% error probability; 0, no significant differences; -, negative differences with < 5% error probability; −−, strong negative differences with < 1% error probability.

*N. striatoreticulatus*	Mean	SD	*O. floridensis* (tightly coiled)	*O. floridensis* (loosely coiled)	*O. soldadensis*	*P. trinitatensis*
First chamber length	454.7	221.33	0	−−	++	++
Proloculus nominal diameter	335.0	151.10	++	++	++	++
Deuteroloculus ratio	1.003	0.1318	0	-	−−	0
Initial marginal radius	360.2	144.63	++	0	++	++
Marginal radius increase	0.062	0.0080	−−	−−	−−	−−
Spiral chamber height increase	3.8	1.45	0	0	++	++
Initial spiral chamber height	112.9	59.18	+	0	++	++
Backbend angle	0.167	0.0527	−−	−−	−−	−−
Initial chamber length	253.2	131.05	0	−−	++	++
Chamber length increase	0.009	0.0064	−−	−−	−−	0
Perimeter ratio	1.102	0.0591	−−	−−	−−	−−

#### Occurrences

Early middle Eocene, P11/12, lower part of Loma Candela Formation; late middle Eocene to late Eocene, NP 16/17, upper part of Loma Candela Formation; late middle Eocene, CNE13/ NP 16, Arroyo Blanco Formation; late Eocene, Jicotea Formation.

#### Remarks

*Nummulites striatoreticulatus* is one of the most widely recognized species of *Nummulites* in the Caribbean. It is distinguished from *N. macgillavry* by the much smaller diameter of the proloculus. In random sections, the range of morphological variation of the species *P. trinitatensis* overlaps with *N. striatoreticulatus* and it is difficult to distinguish between these two species. *Nummulites striatoreticulatus* is rare in the latest Eocene. It is almost absent in the Loma Vigía and Jabaco localities and sparsely present (two specimens) in the Noroña section. These localities represent optimum conditions for orbitoids, with enormous numbers of microspheres and megalospheres of *Lepidocyclina chaperi* and *L. pustulosa*. *Amphistegina cubensis* is less abundant and might replace *N. striatoreticulatus.*

#### Stratigraphical and geographical distribution

Middle Eocene to late Eocece (Lutetian to Priabonian); Cuba, Mexico, Curacao, Florida, Trinidad, Costa Rica, French Lesser Antilles, Panamá, Jamaica and St. Bartheleméy.

Genus ***Palaeonummulites*** Schubert, 1908

#### Type species

*Nummulina pristina* Brady, 1874.

#### Diagnosis

Planispiral, involute, semicompressed to globular; exhibits a tightly to moderately tightly coiled spiral that induces relatively few whorls. Chambers up to twice as high as wide, separated by primary operculine septa. Filaments can be present. The marginal cord is moderately well developed.

Characters and attributes (means and standard deviations) for *Palaeoummulites* and comparison to *Nummulites* and *Operculinoides* are given in Table 4.

**Table 4.** Characters and attributes (means and standard deviations, SD, in mm) for *Palaeoummulites* and comparisons with *Nummulites* and *Operculinoides.* Symbol key: +, differences with < 5% error probability; 0, no significant differences; –, strong negative differences with < 1% error probability.

*Palaeonummulites*	Mean	SD	*Operculinoides*	*Nummulites*
First chamber length	243.3	38.59	−−	−−
Proloculus nominal diameter	113.0	25.84	0	−−
Deuteroloculus ratio	0.888	0.1258	−−	0
Initial marginal radius	140.1	28.76	0	−−
Marginal radius increase	0.094	0.0195	0	0
Spiral chamber height increase	1.7	0.07	−−	−−
Initial spiral chamber height	46.5	10.02	0	−−
Backbend angle	0.495	0.1320	0	+
Initial chamber length	139.0	17.17	−−	−−
Chamber length increase	0.028	0.0175	0	0
Perimeter ratio	1.277	0.1076	0	0

#### Range

Late Paleocene to Recent.

***Palaeonummulites trinitatensis*** (Nuttall, 1928)

(Fig. 17H, I)

1928 *Operculina trinitatensis* Nuttall: 102, pl. 8, figs 10, 11.

1941 *Operculinoides trinitatensis* (Nuttall); Vaughan & Cole: 47, pl. 10, fig. 12, pl. 13, figs 4–14.

1941 *Operculinoides kugleri* (Nuttall); Vaughan & Cole: 18, pl. 10, figs 3–5, 7, 8, pl. 13, figs 1, 2.

1975 *Operculinoides spiralis* (Nuttall); Caudri: 542, pl. 1, fig. 20, pl. 8, fig. 13.

1975 *Operculinoides trinitatensis* (Nuttall); Caudri: 541, pl. 1, figs 10, 16, pl. 8, figs 14, 15.

#### Material

Four megalospheric specimens in equatorial section from Loma Candelaria (98LC-1).

#### Description

 

##### External features

The test is planispiral, involute, laterally slightly compressed. No trace of septal sutures and ornamentation is visible due to poor preservation of the individuals studied here.

##### Internal features

Megalospheric generation with spherical proloculus (mean diameter = 0.1 mm) followed by a reniform deuteroloculus and a moderately tightly coiled spiral with commonly three whorls. Rapid increase in height of the last spiral, with chamber height roughly 3 times greater than chamber length. Operculine primary septa with strong backbend angle gently tapered towards inner ends.

Characters and attributes for *Palaeonummulites trinitatensis* and comparisons with *Nummulites striatoreticulatus*, *Operculinoides floridensis* (tightly coiled), *O. floridensis* (loosely coiled) and *O. soldadensis* are given in Table 5.

**Table 5.** Characters and attributes (means and standard deviations, SD, in mm) for *Palaeonummulites trinitatensis* and comparisons with *Nummulites striatoreticulatus, Operculinoides floridensis* (tightly coiled), *O. floridensis* (loosely coiled) and *O. soldadensis.* Symbol key: ++, strong positive differences with < 1% error probability; +, differences with < 5% error probability; 0, no significant differences; -, negative differences with < 5% error probability; −−, strong negative differences with < 1% error probability.

*P. trinitatensis*	Mean	SD	*O. floridensis* (tightly coiled)	*O. floridensis* (loosely coiled)	*O. soldadensis*	*N. striatoreticulatus*
First chamber length	218.2	53.81	−−	−−	0	−−
Proloculus nominal diameter	99.0	28.91	−−	−−	0	−−
Deuteroloculus ratio	0.950	0.1376	0	−−	-	0
Initial marginal radius	126.8	32.48	−−	−−	0	−−
Marginal radius increase	0.099	0.0172	0	−−	−−	++
Spiral chamber height increase	1.7	0.30	−−	0	0	−−
Initial spiral chamber height	35.5	17.71	−−	−−	0	−−
Backbend angle	0.445	0.1165	0	-	−−	++
Initial chamber length	151.9	26.76	−−	−−	0	−−
Chamber length increase	0.025	0.0149	0	0	−−	0
Perimeter ratio	1.263	0.0859	0	-	−−	+

#### Occurrence

Late middle Eocene to late Eocene NP 16/17, Loma Candela Formation.

#### Remarks

*Palaeonummulites tinitantensis* is not abundant in the Eocene of western and central Cuba but is sporadically present at the Loma Candelaria locality. Cole (1961) admitted that it is difficult to distinguish between *P. trinitatensis* and *P. willcoxi*; the latter is the most widely recognized nummulitid in the Caribbean province and is absent at the studied localities. The lack of an easily recognizable holotype has led to many different morphotypes being described as *Nummulites* or *Operculinoides willcoxi*. We regard the specimen illustrated by Barker (1939) as the most similar to the original description, whereas specimens illustrated in Cole (1941) conform more closely to the moderately tightly coiled *O. floridensis*.

#### Stratigraphical and geographical distribution

Late Eocene (Priabonian); Cuba, Trinidad.

Genus ***Operculinoides*** Hanzawa, 1935

#### Diagnosis

Planispiral, involute or partially involute in the nepionic stage, becoming evolute in the adult stage. Tests with the strongest marginal radius increase and strongest backward bend angles of the investigated individuals, producing rapidly widening coils and highly projecting later chambers. Chambers are up to 4 times as high as wide and are separated by primary operculine septa with septal undulations, which are more pronounced in loosely coiled forms. These forms with the highest values in chamber height in the adult stage have chambers up to 10 times higher than wide. The marginal cord is moderately well developed.

Characters and attributes (means and standard deviations) for *Operculinoides* and comparison to *Nummulites* and *Palaeoummulites* are given in Table 6.

**Table 6.** Characters and attributes (means and standard deviations, SD, in mm) for *Operculinoides* and comparisons with *Nummulites* and *Palaeoummulites.* Symbol key: ++, strong positive differences with < 1% error probability; 0, no significant differences; −−, strong negative differences with < 1% error probability.

*Operculinoides*	Mean	SD	*Palaeonummulites*	*Nummulites*
First chamber length	369.0	165.69	++	0
Proloculus nominal diameter	146.3	67.08	0	−−
Deuteroloculus ratio	1.166	0.1684	++	++
Initial marginal radius	192.0	88.59	0	−−
Marginal radius increase	0.126	0.0191	0	++
Spiral chamber height increase	2.8	1.15	++	−−
Initial spiral chamber height	62.5	32.84	0	−−
Backbend angle	0.636	0.1120	0	++
Initial chamber length	224.0	126.60	++	0
Chamber length increase	0.048	0.0220	0	++
Perimeter ratio	1.392	0.1130	0	++

#### Occurrences

*Operculinoides* is common in the middle and late Eocene.

#### Remarks

Eames *et al*. (1962) included *Operculinoides* Hanzawa, 1935 as a synonym of *Palaeonummulites* based on the type species *Palaeonummulites willcoxi* with a tight coil producing chambers one and half times higher than long, i.e. almost square. This was followed by Haynes (1988), Robinson & Wright (1993) and Haynes *et al*. (2010). However, the other *Operculinoides* species, such as the *O. floridensis* group with a clear operculinid lax coiling and gradational involution, cannot be considered *Palaeonummulites*. It would be necessary to change the type species of *Operculinoides* to distinguish these forms generically. The species *O. floridensis* seems to be the best candidate, as has already been suggested by Butterlin (1981), because the variability of the coiling mode encompasses characteristics of *Palaeonummulites*, *Operculinoides* and *Operculina*.

***Operculinoides floridensis*** (Heilprin, 1885)

(Fig. 16A–H)

**Figure 16.**
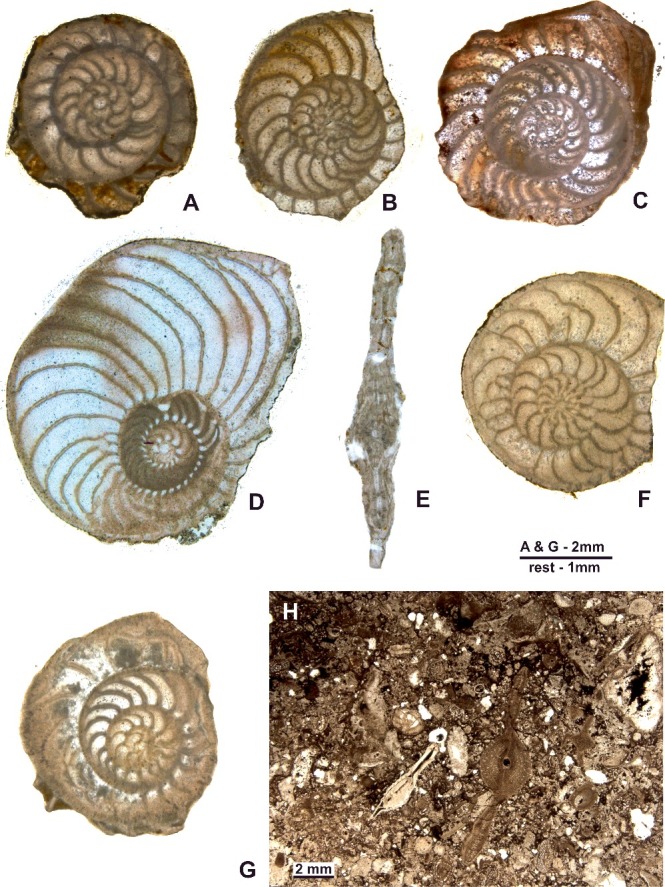
*Operculinoides floridensis* (Heilprin). **A–C,** Loma Candelaria; **A,** 98LC-1-651; **B,** 98LC-1-667; **C,** 98LC-1-815. **D, E,** Loma Vigía; **D,** CA-216-F3-16; **E,** CA-216-D1a. **F,** Loma El Santo, CA-215-852. **G,** Loma Jabaco, LM-52-759. **H,** Angelita Quarry, 98MT-1. **A–D, F** and **G** are A forms in equatorial section; **E** and **F** are A forms in axial section.

1885 *Nummulites floridensis* Heilprin: 321.

1941 *Operculinoides floridensis* (Heilprin); Cole: 20, pl. 9, fig. 8, pl. 10, figs 1–3.

1941 *Operculinoides willcoxi* (Heilprin); Cole: 32, pl.9, figs 2, 3.

1958 *Operculinoides floridensis* (Heilprin); Cole: pl. 33, fig. 2.

1974 *Nummulites (Operculina) floridensis* Heilprin; Frost & Langenheim: 77, pl. 12, figs 1–9.

1981 *Nummulites floridensis* Heilprin; Butterlin: 31, pl. 10, figs 3, 4.

1993 *Palaeonummulites floridensis* (Heilprin); Robinson & Wright: 333, pl. 30, figs 1–3.

#### Material

Twenty-seven megalospheric specimens in equatorial section, comprising 19 from Loma Candelaria (98LC-1), three from Loma El Santo (CA-215), four from Loma Vigía (CA-216) and one from Loma Jabaco (LM-52).

#### Description

 

##### External features

The planispiral test varies from flattened to robust forms. Flattened forms are laterally compressed and fragile, with a prominent, sharply defined umbo due to a partially involute nepionic stage. Robust forms are more involute with a less inflated umbo. External surface smooth or marked by slightly raised septal sutures.

##### Internal features

Megalospheric generations in equatorial section are characterized by a small, spherical to subspherical proloculus with a mean diameter of 0.2 mm followed by a reniform deuteroloculus, then by a variably coiled spiral. The individuals with the highest rates of marginal radius increase (lax variants) have at the adult stage 2–3 rapidly enlarging whorls. Chambers are separated by operculine septa with pronounced septal undulations. Chamber height in the adult stage can be more than 10 times higher than chamber width. The other end of this range of morphological variability is typified by more inflated individuals with a weaker marginal radius increase, producing tightly coiled spirals in which the adult test involves 3–4 whorls. Chamber height is up to 4 times chamber length. Septal undulations are less pronounced.

Characters and attributes (means and standard deviations) for *Operculinoides floridensis* (tightly and loosely coiled) and comparisons with *Nummulites striatoreticulatus*, *Palaeonummulites trinitatensis* and *Operculinoides soldadensis* are given in Tables 7 and 8.

**Table 7.** Characters and attributes (means and standard deviations, SD, in mm) for *Operculinoides floridensis* (tightly coiled) and comparisons with *Nummulites striatoreticulatus*, *Palaeonummulites trinitatensis*, *O. floridensis* (loosely coiled) and *Operculinoides soldadensis.* Symbol key: ++, strong positive differences with < 1% error probability; 0, no significant differences; -, negative differences with < 5% error probability; −−, strong negative differences with < 1% error probability.

*O. floridensis* (tightly coiled)	Mean	SD	*O. floridensis* (loosely coiled)	*O. soldadensis*	*P. trinitatensis*	*N. striatoreticulatus*
First chamber length	431.2	92.32	−−	++	++	0
Proloculus nominal diameter	192.8	44.16	0	++	++	−−
Deuteroloculus ratio	1.080	0.0797	-	0	0	0
Initial marginal radius	255.3	51.51	0	++	++	−−
Marginal radius increase	0.113	0.0132	-	−−	0	++
Spiral chamber height increase	3.7	0.68	0	++	++	0
Initial spiral chamber height	77.1	23.95	0	++	++	-
Backbend angle	0.563	0.1055	0	−−	0	++
Initial chamber length	287.2	78.67	-	++	++	0
Chamber length increase	0.033	0.0117	0	−−	0	++
Perimeter ratio	1.302	0.0758	0	−−	0	++

**Table 8.** Characters and attributes (means and standard deviations, SD, in mm) for *Operculinoides floridensis* (loosely coiled) and comparisons with *Nummulites striatoreticulatus*, *Palaeonummulites trinitatensis*, *O. floridensis*(tightly coiled) and *Operculinoides soldadensis.* Symbol key: ++, strong positive differences with < 1% error probability; +, differences with < 5% error probability; 0, no significant differences; −−, strong negative differences with < 1% error probability.

*O.floridensis* (loose coiled)	Mean	SD	*O.floridensis* (tight coiled)	*O.soldadensis*	*P.trinitatensis*	*N.striatoreticulatus*
First chamber length	655.5	116.47	++	++	++	++
Proloculus nominal diameter	213.6	43.52	0	++	++	−−
Deuteroloculus ratio	1.317	0.1965	+	0	++	+
Initial marginal radius	292.5	50.03	0	++	++	0
Marginal radius increase	0.136	0.0151	+	0	++	++
Spiral chamber height increase	3.2	1.35	0	0	0	0
Initial spiral chamber height	93.0	33.38	0	++	++	0
Backbend angle	0.632	0.0866	0	0	+	++
Initial chamber length	434.4	104.66	+	++	++	++
Chamber length increase	0.042	0.0187	0	0	0	++
Perimeter ratio	1.435	0.1299	0	0	+	++

#### Occurrences

Early middle Eocene, NP14/15, Peñon Formation; late middle Eocene to late Eocene, NP16/17 upper part of Loma Candela Formation; late middle Eocene, CNE13, Arroyo Blanco Formation; late Eocene, NP19-20/CP 15, Jabaco Formation; late Eocene, NP17/19, Blanco Formation.

#### Remarks

*Operculinoides floridensis* is one of the most widely recognized operculinoid species in the Caribbean province. It exhibits a wide range of variability in coiling, which overlaps with the characteristics of *Operculinoides*, *Palaeonummulites* and *Operculina*. The tightly to moderately coiled Cuban specimens are similar to those described by Frost & Langenheim (1974) from Chiapas. Abundant loosely coiled forms were found in localities with optimum conditions for lepidocyclinids and orthophragminids in contrast to localities with *Nummulites striatoreticulatus*. Intra-population morphological diversity is greatest for *O. floridensis* at Loma Candelaria (98LC-2) where tightly to moderately loosely coiled forms occur.

#### Stratigraphical and geographical distribution

Middle Eocene to late Eocene (Lutetian to Priabonian) Cuba, US Gulf Coast, Peru, Curacao, Mexico, Ecuador, Panamá, St. Bartheleméy, Trinidad, Jamaica, Costa Rica, Brazil.

***Operculinoides soldadensis*** Vaughan & Cole, 1941

(Fig.17A–G)

1941 *Operculinoides soldadensis* Vaughan & Cole: 18, pl. 9, figs 5–8, pl. 10, figs 1, 2.

1947 *Nummulites* (*Operculinoides*) *floridensis* (Vaughan & Cole); de Cizancourt: 517, pl. 25, figs 8–10, 13.

1961 *Nummulites floridensis* Heilprin; Butterlin: 12, figs 5–6.

1975 *Operculinoides soldadensis* Vaughan & Cole; Caudri: 537, pl. 1, fig. 11, pl. 8, figs 5–8, 10.

1996 *Operculinoides suteri* Caudri; Caudri: 1189, pl. 10, fig. 9.

#### Material

Twenty-five megalospheric specimens in equatorial section, comprising five from Loma Candelaria (98LC-1), four from Loma El Santo (CA-215), one from Loma Jabaco (LM-52) and 15 from Noroña (NOR-UN).

#### Description

 

##### External features

Test planispiral, flattened, last whorl fragile and laterally compressed, involute in the nepionic stage, becoming evolute in the last whorl. The prominent central umbo is surrounded by slightly raised septal sutures.

##### Internal features

Megalospheric generation with spherical proloculus with a mean diameter of 0.09 mm, followed by reniform deuteroloculus and a loosely coiled spiral with commonly two to three whorls. Rapid increase in height of the last spiral with chamber height roughly 4–5 times higher than chamber width. Primary operculine septa with strong backbend angle gently tapered towards inner ends and with septal undulations. A diagnostic characteristic are the numerous and narrow chambers.

Characters and attributes (means and standard deviations) for *Operculinoides soldadensis* and comparisons with *Nummulites striatoreticulatus*, *Palaeonummulites trinitatensis*, *Operculinoides floridensis* (tightly coiled) and *O. floridensis* (loosely coiled) are given in Table 9.

**Table 9.** Characters and attributes (means and standard deviations, SD, in mm) for *Operculinoides soldadensis* and comparisons with *Nummulites striatoreticulatus*, *Palaeonummulites trinitatensis*, *Operculinoides floridensis* (tightly coiled) and *O. floridensis* (loosely coiled). Symbol key: ++, strong positive differences with < 1% error probability; 0, no significant differences; −−, strong negative differences with < 1% error probability.

*O. soldadensis*	Mean	SD	*O. floridensis* (tightly coiled)	*O. floridensis* (loosely coiled)	*P. trinitatensis*	*N. striatoreticulatus*
First chamber length	252.0	92.62	−−	−−	0	−−
Proloculus nominal diameter	92.2	29.93	−−	−−	0	−−
Deuteroloculus ratio	1.191	0.1834	0	0	+	++
Initial marginal radius	116.9	44.90	−−	−−	0	−−
Marginal radius increase	0.133	0.0193	++	0	++	++
Spiral chamber height increase	2.0	0.96	−−	0	0	−−
Initial spiral chamber height	38.5	15.47	−−	−−	0	−−
Backbend angle	0.687	0.1008	++	0	++	++
Initial chamber length	129.1	43.24	−−	−−	0	−−
Chamber length increase	0.060	0.0202	++	0	++	++
Perimeter ratio	1.448	0.0809	++	0	++	++

#### Occurrences

Middle late Eocene to late Eocene, NP 16/ NP17, upper part of the Loma Candela Formation; late middle Eocene, CNE13, Arroyo Blanco Formation; ?early Oligocene O1/P18 and NP 21 /CP 16, Jabaco Formation.

#### Remarks

Cole (1958) considered *O. soldadensis* to be synonymous with *O. floridensis*; however, our morphometric analysis based on growth-independent and growth-invariant characters clearly distinguished the two species (Table 3): *Operculinoides soldadensis* shows fewer morphological variations (ecophenotypes) at distinct depositional gradients than *O. floridensis*.

#### Stratigraphical and geographic dialstribution

Middle to late Eocene (Lutetian to Priabonian); Cuba Trinidad, Mexico.

***Operculinoides ocalanus*** (Cushman, 1921)

(Fig. 17J)

**Figure 17.**
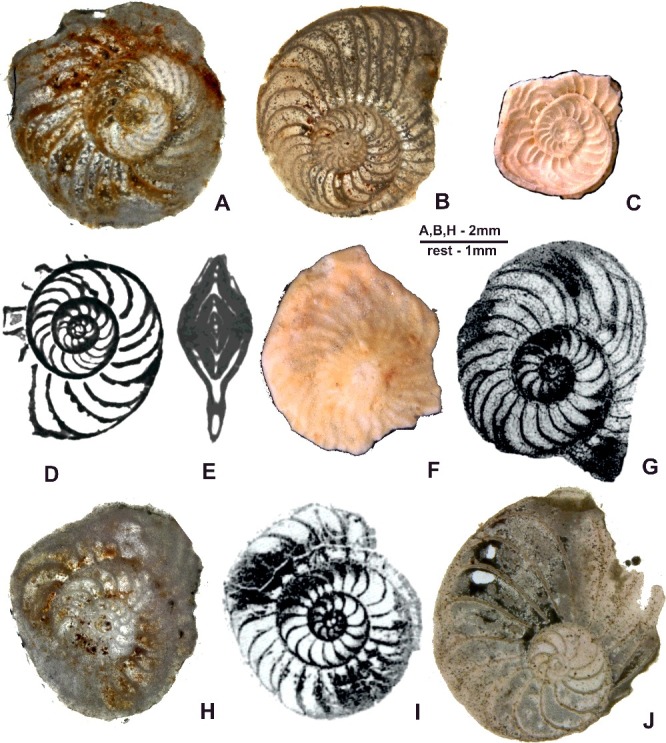
**A–G,** *Operculinoides soldadensis* Vaughan & Cole; **A,** Loma El Santo, CA-215-871; **B,** Loma Candelaria, 98LC-1-669; **C–F,** Noroña; **C,** NOR-UN 24; **D–F,** NOR-UN 15/14; **G,** holotype, Trinidad. **H, I,** *Palaeonummulites trinitatensis* (Nutall); **H,** Loma Candelaria, 98LC-1ICT3; **I,** holotype of *Operculinoides kugleri* Vaughan & Cole, Trinidad. **J,** *Operculinoides ocalanus* (Cushman), Loma Jabaco, CA-4-724. **A–D, G–J,** A forms in equatorial section; **E,** A form in axial section; **F,** external view.

1921 *Operculina ocalana* Cushman: 129, pl. 19, figs 4, 5.

1941 *Operculinoides ocalanus* (Cushman); Vaughan & Cole: 38, pl. 8, figs 8, 9, pl. 9, figs 1–4, pl. 10, fig. 1.

1975 *Operculinoides ocalanus* (Cushman); Caudri: 537, pl. 1, fig. 12, pl. 8, figs 4, 9.

1996 *Operculinoides ocalanus* (Cushman); Caudri: 1187, pl. 5, fig. 5, pl. 9, figs 11–13.

#### Material

Two megalospheric specimens in equatorial section from Loma Jabaco (LM-52).

#### Occurrence

Late Eocene, NP19-20/CP 15, Jabaco Formation.

#### Stratigraphical and geographical distribution

Middle Eocene to late Eocene (Lutetian to Priabonian); Cuba, Florida, Trinidad.

Genus ***Heterostegina*** d'Orbigny, 1826

#### Type species

*Heterostegina depressa* d‘Orbigny, 1826.

#### Remarks

Differences between species of *Heterostegina* are given in Torres-Silva et al. (2017, table 3).

***Heterostegina cubana*** Cizancourt, 1947

(Fig. 18C)

**Figure 18.**
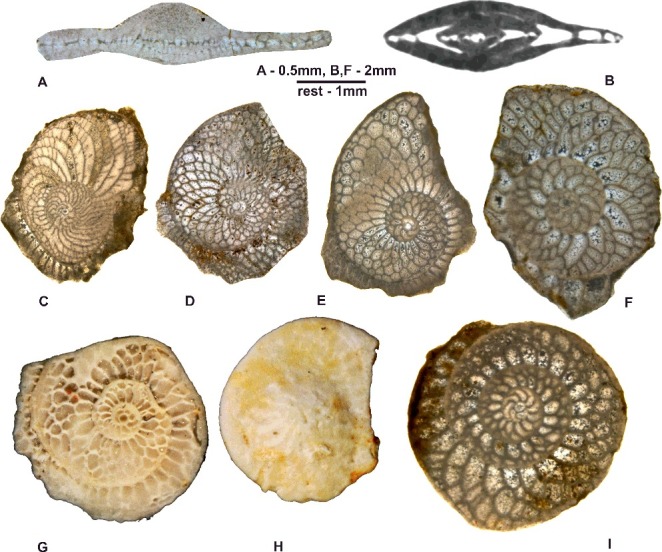
**A, B, D–H,** *Heterostegina ocalana* Cushman; **A,** Loma Vigía, CA-216-D1a; **B,** Noroña, NOR-UN 15/14; **D,** Loma Vigía, CA-216-79; **E, F,** Loma Jabaco; **E,** LM-52-756; **F,** LM-52-752; **G, H,** Noroña, NOR-UN 24. **C,** *Heterostegina cubana* Cizancourt, Loma candelaria, 98LC-1H-809. **I,** *Heterostegina* sp. indet., Loma Candelaria, 98LC-1H-808. **A, B,** A forms in axial section; **C–G, I,** A forms in equatorial section; **H,** external view.

1947 *Heterostegina cubana* de Cizancourt: 518, pl. 25, figs 4, 5.

2017 *Heterostegina cubana* de Cizancourt; Torres-Silva, Hohenegger, Ćorić, Briguglio, & Eder: 57, fig. 10E.

#### Material

Ten megalospheric specimens in equatorial section and numerous random thin sections from Loma Candelaria section (98LC-1).

#### Description

 

##### External features

Test involute, flattened, biconvex, with diameter of the megalospheric forms ranging from 4.1 to 5.9 mm. The piles appear more pronounced near the central portion of the test. No trace of septal sutures and secondary chamberlets is visible due to bad preservation of the individuals at the Loma Candelaria locality. Cizancourt's (1947) original description is based on specimens with a granulate surface and primary and secondary septa forming the typical reticulate network in *Heterostegina*, and with septal sutures slightly curved towards the periphery.

##### Internal features

Megalospheric generation characterized by large mean proloculus diameter value (0.25 mm) followed by a second reniform chamber and by a rapidly increasing, loosely coiled spiral. Primary septa with stronger backwards bend angle form elongated chambers, which increase in height during ontogeny. After the second chamber, one to five operculinid chambers (undivided chambers) are followed by chambers subdivided into chamberlets by very incompletely developed secondary septa or septula. The first chamberlet closest to the marginal spiral is extremely elongated compared to peripheral chamberlets. Chambers subdivided by complete septula form rectangular chamberlets.

#### Occurrences

Late middle Eocene to late Eocene, NP 16/17, Loma Candela Formation.

#### Remarks

*Heterostegina cubana* was first described from the late Eocene of western Cuba by Cizancourt (1947) and was almost unrecorded until Cole (1957) considered it a synonym of *Heterostegina ocalana*. This species is distinguished by its characteristic incomplete septula and larger proloculus. Caudri (1996) reported *Heterostegina indicata* with very incompletely developed or absent septa in the basal late Eocene of Trinidad. As suggested by Caudri (1996) for Trinitarian species, *H. cubana* could also be a transitional form between operculinid and heterosteginid morphologies. Note, however, that *H. indicata* has a complete evolute enrolment similar to *Planostegina* and *Planoperculina*, whereas *H. cubana* shows a distinct thickening of the central test.

#### Stratigraphical and geographical distribution

Late middle Eocene to late Eocene (Bartonian to early Priabonian); Cuba.

***Heterostegina ocalana*** Cushman, 1921

(Fig. 18A, B, D–H)

1921 *Heterostegina ocalana* Cushman, 130, pl. 21, figs 15–18.

1941 *Heterostegina ocalana* Cushman; Cole: 32, pl. 11, figs 3–6.

1952 *Heterostegina ocalana* Cushman; Cole: 13, pl. 4, figs 2–18.

1957 *Heterostegina ocalana* Cushman; Puri: 136, pl. 6, figs 10, 11, pl. 7, fig. 16.

1993 *Heterostegina (Vlerkina) ocalana* Cushman; Robinson & Wright: 335, pl. 31, fig. 4.

2017 *Heterostegina ocalana* Cushman; Torres-Silva, Hohenegger, Ćorić, Briguglio, & Eder: 57, fig. 10B–D.

2017 *Heterostegina ocalana* Cushman; Benedetti, Less, Parente, Pignatti, Cahizac, Torres-Silva, & Buhl: 14, fig. 10A–G.

#### Material

Twenty-seven megalospheric specimens in equatorial sections, comprising 14 from Loma Vigía (CA-216), nine from Loma Jabaco (LM-52) and four from Norona (NOR-UN).

#### Description

 

##### External features

The test is involute, becoming evolute in the last whorls, lenticular to flat, biconvex, and thin towards the periphery with oval contour. Tests of the megalospheric form range in diameter from 1.5 to 4.5 mm. The distinct central pile is situated near the embryonic chambers. The septal sutures are slightly curved, and towards the periphery the primary and secondary sutures form a characteristic reticulate network; this ornamentation is absent in the Loma Vigia populations. B forms are very rare and no significant difference was observed in the size of adults between the megalospheric and microspheric forms.

##### Internal features

Megalospheric generations in equatorial section are characterized by a small and subspherical proloculus 0.067–0.19 mm in diameter (mean 0.14 mm), followed by a second reniform chamber and a loosely coiled spiral. The number of post-embryonic undivided chambers ranges from two to four and they do not reappear after the first heterosteginid chamber. Primary septa, with weaker backbend angle, form arched chambers, subdivided into subrectangular chamberlets by complete septula. The first chamberlets along the inner spiral cord are 2 times wider than the others. The number of chamberlets and septula increases through ontogeny. A second megalospheric morphotype was found with proloculus size between 0.05 and 0.06 mm followed by 6–7 operculinid chambers, confirming the results of Eder *et al*. (2017a) on the extant *Heterostegina depresa* showing strong variability in both characters based on the mixture of two megalospheric generations. Morphological variability in *H. ocalana* has already been published by Cole (1953).

#### Occurrences

Late Eocene, NP17/19, Blanco Formation; late Eocene, NP19-20/CP 15 and ?early Oligocene, O1/P18 and NP 21/CP 16, Jabaco Formation.

#### Remarks

As the most widely recorded heterostegenid species in the American-Caribbean late Eocene, *Heterostegina ocalana* is distinguished by its small proloculi and the great variability in the number of operculinid chambers within specimens from different localities (Cole 1952; Torres-Silva *et al*. 2017). Occurrences of *H. ocalana* at Dowling Park (Florida) dated by strontium isotope stratigraphy correspond to the latest Priabonian, roughly fitting the E16 planktonic foraminiferal biozone and NP21 calcareous nanofossil zone (Benedetti *et al*. 2017). This is also consistent with the possible early Rupelian age of samples with *H. ocalana* from the Norona section, in which this species appears to be significantly more highly evolved than the Priabonian specimens elsewhere.

#### Stratigraphical and geographical distribution

Late Eocene (Priabonian); Cuba, Florida, Panama, Jamaica, and Island of the Grenadines.

***Heterostegina*** sp. indet.

(Fig. 18I)

2017 *Heterostegina* sp. indet. Torres-Silva, Hohenegger, Ćorić, Briguglio, & Eder: 57, fig. 10F.

#### Material

Three megalospheric specimens in equatorial section from Loma Candelaria (98LC-1).

#### Description

This species is known only from its internal equatorial morphology. Test diameter ranges from 3.1 to 3.5 mm. The embryonic apparatus is characterized by a large and subspherical proloculus between 0.2 and 0.25 mm wide. It is followed by a second chamber of similar in size and form, and by a spiral with a weaker marginal radius increase. It produces many whorls, with shorter chambers; hence, it shows a decrease in the number of chamberlets in comparison to *H. cubana* and *H. ocalana.* Primary septa have a weaker backbend angle in the first few whorls, hence forming straighter chambers. In later whorls, the chambers became more arched. After the embryonic stage, two undivided chambers are followed by chambers that are subdivided into sub-rectangular to hexagonal chamberlets, divided by complete septula. The first chamberlet nearest to the marginal spiral is 2–3 times longer than the others. The number of chamberlets and septula increases during ontogeny.

#### Remarks

This seems to be a new species because it is significantly distinct from *H. ocalana* and *H. cubana* in its marginal radius increase and the size and form of the embryonic apparatus. The latter is the most important character for species delimitation, especially in larger benthic foraminifera. Due to missing axial sections, the possibility that this new species might actually be *Spiroclypeus* cannot be rejected.

#### Occurrences

Late middle Eocene to late Eocene, NP 16/17, Loma Candela.

## Discussion

### Palaeoenviromental implications and evolutionary trends

After determining four species based on growth-independent and growth-invariant characters, significant morphological variations within species were detected using discriminant analyses. In order to distinguish the source of morphological changes, the results of the discriminant analyses were coupled with the stratigraphical and palaeoenvironmental interpretations of the different localities. Morphological variants within species along palaeoecological gradients (localities) are interpreted as ecophenotypes. Morphological changes within species between locations at different stratigraphical levels but representing similar palaeoecological conditions are interpreted as evolutionary trends, as the environmental impact on morphology at these localities should be of similar magnitude.

**Figure 19.**
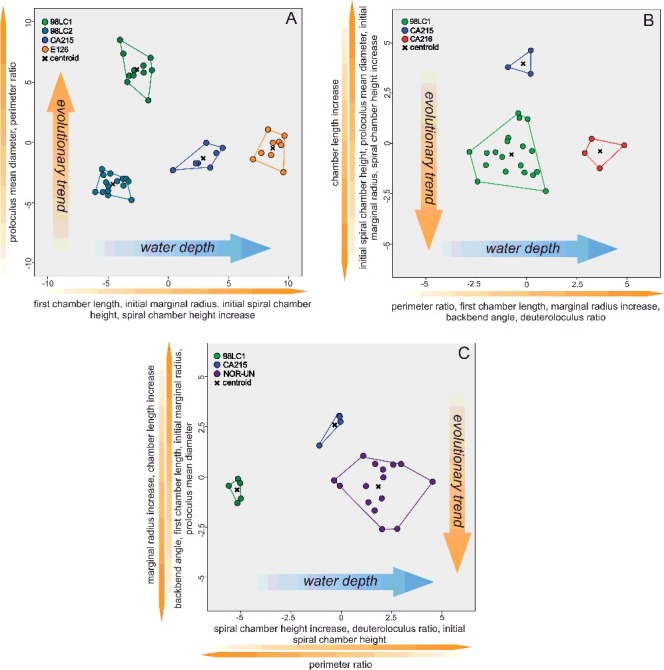
Discriminant analysis of nummulitid species, where the important discriminators are ranked along the discriminant functions. Orange arrows indicate possible source of morphological changes. **A,** discriminant analysis within *Nummulites striatoreticulatus* at localities 98LC-2, 98LC-2 and E-126. **B,** discriminant analysis within *Operculinoides floridensis* at localities 98 LC-1, CA-215 and CA-216. **C,** discriminant analysis within *O. soldadensis* at localities 98LC-1, CA-125 and NOR-UN.

The first axis of discriminatory analysis in Figure 19 clearly documents, in all species, the palaeoecological separation of populations. The second discriminant axis apparently reflects evolutionary tendencies. Nummulitid test morphology and palaeoenvironmental gradients were compared with those of extant related nummulitid groups, assuming an analogous morphological response to palaeoenvironments in the fossils forms.

Comparing *Nummulites striatoreticulatus* individuals across Entronque de Herradura (98LC-2), Loma Candelaria (98LC-1), Loma El Santo (CA-215) and Jicotea (E-126) clearly shows the strongest variations in morphology. These variations are related to parameters characterizing the embryonic apparatus. The increases in values of first chamber length (FCL), initial marginal radius (IMR) and initial spiral chamber height (ICH), but also spiral chamber height increase (CHInc), are apparently related to a deepening of the environment or in the source of the carbonates (Fig. 19A). A steady increase of the proloculus mean diameter (PD) and perimeter ratio (PerP) over time is expressed in the second axis. The strongest differences in these characters were found between specimens from Entronque de Herradura (98LC-2) and those from Loma Candelaria (98LC-1) (Fig. 19A). The increase in proloculus size and elongation of equatorial chambers through the Lutetian until the early Priabonian might be interpreted as apparent time-dependent evolutionary trends because the two localities show similar palaeoecological conditions. They represent parts of the continuous sedimentation process of the Loma Candela Formation. A well-documented trend of increasing proloculus size over time has been considered one of the most noticeable evolutionary tendencies in *Nummulites* (e.g. Schaub 1963, 1981; Blondeau 1972; Samanta 1981; Cotton *et al*. 2016).

All megalospheric generations of the species *O.* gr*. floridensis* show a significant depth trend, becoming flatter with increasing depth. This trend is well documented between the localities Loma Candelaria (98LC-1) and Loma Vigia (CA-216), representing distinct transects along a depositional gradient (Fig. 19B). The transition from the moderately coiled forms at Loma Candelaria to loosely coiled forms at Loma Vigía is manifested by higher values of perimeter ratio (PerP), first chamber length (FCL), marginal radius increase (MRInc), backbend angle (BBA) and deuteroloculus ratio (DW) in the flatter individuals (Fig. 18B). Note that extant *Operculina* species show significantly increasing radius expansion rates with increasing water depth, at least in the megalospheric generation (Yordanova & Hohenegger 2004).

Individuals from the Loma Candelaria (98LC-1) and Loma El Santo (CA-215) localities do not show lesser differences along the axis representing palaeoecological influence, as both locations are characterized by tightly coiled forms of *O. floridensis* co-occurring with *N. striatoreticulatus.* The strongest differences between these localities seem to be due to the decrease of the initial spiral chamber height (CH), proloculus nominal diameter (PD), initial marginal radius (IMR) and spiral chamber height increase (CHInc), and the higher values of chamber length increase (CLInc) (Fig 19B). These differences could be tentatively interpreted as stratigraphically influenced because Loma El Santo (CA-215) ranges from middle to late Lutetian and Loma Candelaria (98LC-1) ranges from Bartonian to early Priabonian. The localities Loma Candelaria (98LC-1) and Loma Vigia (CA-216) show fewer differences along the second axis, as expected from their stratigraphical proximity (Fig 19B).

Unlike *O. floridensis*, which extends over a range of coiling that nearly overlaps the variability of *Palaeonummulites* and *Operculinoides*, *O. soldadensis* represents a typical *Operculinoides* with low variability in the expansion of whorl height. Because *O. soldadensis* already starts at its shallowest occurrence (Loma Candelaria) with a high marginal radius increase, the phenotypic correlation between marginal radius increase and water depth is much weaker in *O. soldadensis* than in *O. floridensis*, which is similar to recent *Planostegina* species (Yordanova & Hohenegger 2004). The marginal radius increase (MRInc) and chamber length increase (CLInc) are stratigraphically important. Higher values are characteristic for late Eocene individuals (Fig. 19C). The decrease through time in backbend angle (BBA), first chamber length (FCL), initial marginal radius (IMR) and proloculus nominal diameter (PD) is also stratigraphically significant (Fig 19C). Characters potentially affected by water depth are spiral chamber height increase (CHInc), deuteroloculus ratio (DW), initial spiral chamber height (CH) and perimeter ratio (PerP) (Fig. 19C). Septal undulations are a characteristic trait in both *Operculinoides* species from deeper environments. In recent *Operculina complanata* it is a strategy to strengthen the test of the strongly flattened late whorls (Hohenegger 2011a).

Unlike *Nummulites* sensu stricto, proloculus size in *Operculinoides* exhibits a negative trend with a diameter decrease through time. Nonetheless, the present results demonstrate that proloculus size in *O. floridensis* is only of moderate importance, and in *O. soldadensis* of the lowest importance, as a separator for evolutionary trends within species. Similar results have been detected in *Heterostegina* populations from the Cuban Eocene (Torres-Silva *et al*. 2017). Therefore, at least in those nummulitids non-taxonomically grouped as operculinid foraminifera (Hottinger 1977), our results do not support changes in proloculus size as a key morphological indicator of evolutionary changes. The stratigraphical trends towards the late Eocene with higher frequencies of flatter tests coincide with the extinction at the middle/late Eocene boundary of most large species of *Nummulites*, possibly related to a decrease in oceanic temperature (Shackleton & Kennet 1975; Bohaty *et al*. 2009) or a transgression at the Bartonian/Priabonian boundary (Miller *et al*. 1998, 2011).

Based on palaeoenvironment reconstructions and on the occurrence of each species at different localities, we infer approximate palaeodepth ranges (Fig. 7). Most deposited LBF assemblages, such as recent dead assemblages and fossil taphonocoenoses, represent a mixture of autochthonous and allochthonous specimens, thus compromising the recognition of the ecological optimum zone of a species (Hohenegger & Yordanova 2001; Yordanova & Hohenegger 2002; Briguglio & Hohenegger 2011). Since morphometric data within the localities show no major outliers, this hints at autochthonous populations at Entronque de Herradura, Loma Candelaria and Loma Vigia. This is additionally reflected by facies analysis. Interpreting the sediments in which nummulitid populations were found (Noroña, Loma Jabaco and Loma el Santo) implies some degree of allochthony, but without a significant mixing of individuals from different depths. *Operculinoides floridensis* and *O. soldadensis* show a broader variability in marginal radius increase (MRInc) and thus probably occupied wider niches than *N. striatoreticulatus*. The latter seems to have been restricted to the shelf edge and to the shallowest parts of the upper slope. Similar palaeoecological ranges have been noted in the Jamaican Eocene (Robinson 2004). Highest nummulitid diversity was found at locality 98LC-1, representing the shelf edge or the shallowest parts of the upper slope.

### Phylogenetic inferences

The opportunity to test possible phylogenetic connections between operculines and heterostegines is provided by the comparison of the morphometric and biostratigraphical approach used here to the results on Eocene *Heterostegina* from Cuba (Torres-Silva *et al*. 2017). It has been suggested that *Heterostegina* evolved from different species of *Operculina* in different lineages at various times (Hottinger 1977; Herb 1978; Banner & Hodgkingson 1991; Lunt & Renema 2014). However, fully evolute *Operculina* species are uncommon in the Caribbean Eocene, with erratic appearances in the late Eocene (Butterlin 1981; Caudri 1996). Unlike *Operculina*, involute to semi-involute *Operculinoides* species are widespread in the American-Caribbean province and are more likely to be ancestors because their transition between involute and evolute enrolment is much more similar to *Heterostegina*. Morphological relationships between the operculine and heterostegine species from the Cuban localities are suggested based on PCA (Fig. 5A, B). The earliest members of the *Heterostegina* lineages occur in the Bartonian to early Priabonian (NP16–17), represented by *Heterostegina cubana* and *H*. sp. indet. Of these species, *H.* sp. indet. exhibits the closest equatorial morphology to tightly coiled forms of *Operculinoides floridensis.* Discriminant analysis documents the strongest similarities in perimeter ratio (PerP), backbend angle (BBA), initial marginal radius (IMR) and proloculus mean diameter (PD) (Fig. 5C). Torres-Silva *et al*. (2017) pointed out closer phylogenetic connections of *H*. sp. indet. to late Priabonian *H. ocalana* than to *H. cubana* based on similarities in the characters perimeter ratio (PerP) (of chamberlets), proloculus nominal diameter (PD), initial spiral chamber height (ICH) and chamberlet length decrease. Transitional forms with incomplete secondary septa such as *H. cubana* appear to have developed independently along parallel lines at different times, such as *H. indicata* Caudri from the late Eocene of Trinidad and *H. heterostegina* (Silvestri) from the early Miocene of Italy. Similar parallel evolution has been shown for recent *Planoperculina* and *Planostegina* by molecular genetic evidence. Despite their great similarity to extant *H. depressa* in equatorial section, no direct genetic relation can be proven (Holzmann *et al*. 2003).

## Conclusions

Current nummulitid taxonomy in the Caribbean province has combined a broad range of coiling including characteristics of *Nummulites*, *Palaeonummulites*, *Operculinoides* and *Operculina* within *Nummulites* sensu lato (e.g. Cole in Loeblich & Tappan 1964; Frost & Langenheim 1974; Butterlin 1981; Robinson 2004). The generalized reconstruction of nummulitid tests in equatorial section presented here allows classification based on the similarities of 11 growth-independent and growth-invariant characters. Analyses distinguished three genera: *Nummulites*, *Paleonummulites* and *Operculinoides*. At the species level, the classification resulted in the partitioning of the Eocene specimens into the four species: *N. striatoreticulatus*, *P. trinitatensis*, *O. floridensis* group and *O. soldadensis*. The main morphological separators between species are the backbend angle (BBA), marginal radius increase (MRInc), perimeter ratio (PerP) and first chamber length (FCL). The separation of *Nummulites* sensu lato into *Palaeonummulites*, *Operculinoides* and *Nummulites* sensu stricto proves the biostratigraphical value of this analysis because species of *Nummulites* sensu stricto occur only from the middle Eocene to early late Eocene, while moderately to loosely coiled operculinid forms have longer stratigraphical ranges from the earliest middle Eocene to probably the early Oligocene. The particular success of loosely coiled, lax forms in the latest Eocene may be coupled with environmental disturbances (e.g. climatic change or eustatic sea level changes) around the middle/late Eocene boundary that caused the extinction of widespread and long-ranging LBF species (Hallock *et al*. 1991; Less & Özcan 2012).

Although morphometric quantification enables the recognition of four species in the Cuban Eocene with sometimes broad morphological variability, the causes of this variability are difficult to explain. The succession of different morphotypes either represents a response to palaeoenviromental influences or reflects time-dependent evolutionary tendencies. The tightness/laxity of the spiral is an important morphological separator at the genus level, but a clear general trend can be coupled with changes in palaeodepth. Since extant Nummulitidae adapt to water depth by test flattening and wall thinning, which are influenced by light intensity and water energy, the same factors can be expected in fossil forms (Hohenegger 1999; Beavington-Penney & Racey 2004; Yordanova & Hohenegger 2004). The significance of nummulitids as palaeoenviromental indicators is supported by the correlation of high frequencies in involute, tightly coiled forms of *N. striatoreticulatus*, *P. trinitatensis* and *O. floridensis* with lower values in marginal radius increase (MRInc). This enables determination of the shelf edge and the shallowest parts of the upper slope environments (Fig. 14B). The transition from tightly to loosely coiled forms of *O. floridensis*, regarded as ecophenotypes, demonstrates that groups within a single species vary significantly in many parameters due to palaeoenvironmental conditions. The correlation in high frequencies of loosely coiled forms, such as *O. floridensis*, *O. soldadensis* and *O. ocalanus*, with the highest values of marginal radius increase (MRInc) coincides with localities representing the deepest parts of the photic zone.

Evolutionary, time-dependent trends within species can be inferred in sections with similar palaeoecological conditions. An increase in proloculus size was detected in *Nummulites striatoreticulatus* from the middle Eocene to late Eocene, supporting this important evolutionary pattern in *Nummulites* sensu stricto. Hence, many species are currently defined by the size increase in proloculus diameter (e.g. Schaub 1981). Further, the strong morphological differences between specimens of successive stratigraphical levels (e.g. Loma Candela Formation) indicate a higher evolutionary rate in *Nummulites* sensu stricto. Operculinid forms showed an opposite and much weaker time-dependent trend in proloculus size decrease. Similar trends have been detected in Eocene *Heterostegina* populations from the same localities (Torres-Silva *et al*. 2017). The evidence of two megalospheric morphotypes in *H. ocalana* (Loma Jabaco) with strong variation in proloculus size and operculinid chambers shows that trends in size and form of the nepiont within a species often depend on (palaeo)environmental gradients (Biekart *et al*. 1985) or (palaeo)biogeographical distribution (Eder *et al*. 2017). In the fossil record this can lead to misinterpretation as evolutionary trends. A phylogenetic connection between *Heterostegina* and *Operculinoides* is suggested by a similar equatorial morphology, based mostly on perimeter ratio (PerP) and backbend angle (BBA), while similarities in parameters characterizing the embryonic apparatus position it closer to *O. floridensis*.

Finally, our morphometric approach has reaffirmed Cole's ideas (1957, 1958) that there are only a few Eocene species in the American-Caribbean province and that these are characterized by high morphological variability, long stratigraphical ranges, and palaeogeographical distributions restricted to this region. His suggestion to increase awareness of the morphological diversity inherent in individual populations and between populations in scientific studies of the Caribbean nummulitids is supported.

## Supplementary Material

Supplemental_headings.docx

Anova_Species.docx

Anova_Genera.docx

CDA_and_PC_loadings.docx

repository.docx
