# Supplementary material for: Morphometric analysis of Eocene nummulitids in western and central Cuba: taxonomy, biostratigraphy and evolutionary trends
Source: J Syst Palaeontol. 2018 Apr 13;17(7):557–95. doi: 10.1080/14772019.2018.1446462 (PMC6474738; doi:10.1080/14772019.2018.1446462)
Supplement: Supplemental_headings.docx [file TJSP_A_1446462_SM8336.docx]

**Appendix 1.** Results of the analysis of variance on genera level.

**Appendix 2.** Results of the analysis of variance on species level.

**Appendix 3.** Results of the CDA analysis and PCA loadings.

**Appendix 4.** Distribution of the nummulitid specimens in the studied sections.
