# Supplementary material for: Morphometric analysis of Eocene nummulitids in western and central Cuba: taxonomy, biostratigraphy and evolutionary trends
Source: J Syst Palaeontol. 2018 Apr 13;17(7):557–95. doi: 10.1080/14772019.2018.1446462 (PMC6474738; doi:10.1080/14772019.2018.1446462)
Supplement: Anova_Species.docx [file TJSP_A_1446462_SM8331.docx]

| **ANOVA species** | | | | | | | | | | | |
| --- | --- | --- | --- | --- | --- | --- | --- | --- | --- | --- | --- |
|  | | | | Sum of Squares | | df | | Mean Square | | F | Sig. |
| FCL | | Between Groups | | 1679410.1 | | 4 | | 419852.5 | | 16.94 | 0.000 |
|  |  | Within Groups | | 2601984.2 | | 105 | | 24780.8 | |  |  |
|  |  | Total | | 4281394.3 | | 109 | |  | |  |  |
| PD | | Between Groups | | 1158649.6 | | 4 | | 289662.4 | | 29.11 | 0.000 |
|  |  | Within Groups | | 1044970.3 | | 105 | | 9952.1 | |  |  |
|  |  | Total | | 2203619.9 | | 109 | |  | |  |  |
| DR | | Between Groups | | 1.3 | | 4 | | 0.3 | | 15.04 | 0.000 |
|  |  | Within Groups | | 2.2 | | 105 | | 0.0 | |  |  |
|  |  | Total | | 3.5 | | 109 | |  | |  |  |
| IMR | | Between Groups | | 1156031.4 | | 4 | | 289007.8 | | 29.87 | 0.000 |
|  |  | Within Groups | | 1015950.1 | | 105 | | 9675.7 | |  |  |
|  |  | Total | | 2171981.5 | | 109 | |  | |  |  |
| MRInc | | Between Groups | | 0.1 | | 4 | | 0.0 | | 141.56 | 0.000 |
|  |  | Within Groups | | 0.0 | | 105 | | 0.0 | |  |  |
|  |  | Total | | 0.1 | | 109 | |  | |  |  |
| CBIn | | Between Groups | | 77.6 | | 4 | | 19.4 | | 14.56 | 0.000 |
|  |  | Within Groups | | 139.9 | | 105 | | 1.3 | |  |  |
|  |  | Total | | 217.5 | | 109 | |  | |  |  |
| ICB | | Between Groups | | 112562.1 | | 4 | | 28140.5 | | 16.67 | 0.000 |
|  |  | Within Groups | | 177274.3 | | 105 | | 1688.3 | |  |  |
|  |  | Total | | 289836.3 | | 109 | |  | |  |  |
| BBA | | Between Groups | | 5.6 | | 4 | | 1.4 | | 189.74 | 0.000 |
|  |  | Within Groups | | 0.8 | | 105 | | 0.0 | |  |  |
|  |  | Total | | 6.4 | | 109 | |  | |  |  |
| ICL | | Between Groups | | 837806.8 | | 4 | | 209451.7 | | 22.01 | 0.000 |
|  |  | Within Groups | | 999127.4 | | 105 | | 9515.5 | |  |  |
|  |  | Total | | 1836934.2 | | 109 | |  | |  |  |
| CLInc | | Between Groups | | 0.0 | | 4 | | 0.0 | | 60.20 | 0.000 |
|  |  | Within Groups | | 0.0 | | 105 | | 0.0 | |  |  |
|  |  | Total | | 0.1 | | 109 | |  | |  |  |
| PerR | | Between Groups | | 2.4 | | 4 | | 0.6 | | 98.03 | 0.000 |
|  |  | Within Groups | | 0.6 | | 105 | | 0.0 | |  |  |
|  |  | Total | | 3.0 | | 109 | |  | |  |  |
| **Test of Homogeneity of Variances** | | | | | | | | |  |  |  |
|  | Levene Statistic | | df1 | | df2 | | Sig. | |  |  |  |
| FCL | 12.664 | | 4 | | 105 | | .000 | |  |  |  |
| PD | 39.587 | | 4 | | 105 | | .000 | |  |  |  |
| DR | 4.272 | | 4 | | 105 | | .003 | |  |  |  |
| IMR | 15.607 | | 4 | | 105 | | .000 | |  |  |  |
| MRInc | 8.186 | | 4 | | 105 | | .000 | |  |  |  |
| CBIn | 7.674 | | 4 | | 105 | | .000 | |  |  |  |
| ICB | 11.417 | | 4 | | 105 | | .000 | |  |  |  |
| BBA | 4.733 | | 4 | | 105 | | .001 | |  |  |  |
| ICL | 7.413 | | 4 | | 105 | | .000 | |  |  |  |
| CLInc | 10.247 | | 4 | | 105 | | .000 | |  |  |  |
| PerR | 4.079 | | 4 | | 105 | | .004 | |  |  |  |

| **Multiple Comparisons** | | | | | | | |
| --- | --- | --- | --- | --- | --- | --- | --- |
| Tamhane |  |  |  |  |  |  |  |
| Dependent Variable | | | Mean Difference (I-J) | SE | Sig. | 95% Confidence Interval | |
|  |  |  |  |  |  | Lower Bound | Upper Bound |
| FCL | O.flor (t) | O.flor (l) | -224.3 | 41.98 | 0.001 | -362.41 | -86.20 |
|  |  | O.sold | 179.2 | 26.69 | 0.000 | 100.46 | 257.89 |
|  |  | P.trin | 213.0 | 27.71 | 0.000 | 126.77 | 299.17 |
|  |  | N.stri | -23.5 | 39.31 | 1.000 | -137.68 | 90.66 |
|  | O.flor (l) | O.flor (t) | 224.3 | 41.98 | 0.001 | 86.20 | 362.41 |
|  |  | O.sold | 403.5 | 40.78 | 0.000 | 267.04 | 539.92 |
|  |  | P.trin | 437.3 | 41.45 | 0.000 | 298.44 | 576.11 |
|  |  | N.stri | 200.8 | 49.96 | 0.004 | 48.29 | 353.30 |
|  | O.sold | O.flor (t) | -179.2 | 26.69 | 0.000 | -257.89 | -100.46 |
|  |  | O.flor (l) | -403.5 | 40.78 | 0.000 | -539.92 | -267.04 |
|  |  | P.trin | 33.8 | 25.85 | 0.900 | -47.40 | 114.99 |
|  |  | N.stri | -202.7 | 38.02 | 0.000 | -313.14 | -92.24 |
|  | P.trin | O.flor (t) | -213.0 | 27.71 | 0.000 | -299.17 | -126.77 |
|  |  | O.flor (l) | -437.3 | 41.45 | 0.000 | -576.11 | -298.44 |
|  |  | O.sold | -33.8 | 25.85 | 0.900 | -114.99 | 47.40 |
|  |  | N.stri | -236.5 | 38.75 | 0.000 | -350.48 | -122.48 |
|  | N.stri | O.flor (t) | 23.5 | 39.31 | 1.000 | -90.66 | 137.68 |
|  |  | O.flor (l) | -200.8 | 49.96 | 0.004 | -353.30 | -48.29 |
|  |  | O.sold | 202.7 | 38.02 | 0.000 | 92.24 | 313.14 |
|  |  | P.trin | 236.5 | 38.75 | 0.000 | 122.48 | 350.48 |
| PD | O.flor (t) | O.flor (l) | -20.8 | 16.80 | 0.929 | -74.27 | 32.76 |
|  |  | O.sold | 100.6 | 11.17 | 0.000 | 67.12 | 134.11 |
|  |  | P.trin | 93.8 | 14.05 | 0.000 | 49.52 | 138.07 |
|  |  | N.stri | -142.2 | 24.98 | 0.000 | -215.07 | -69.33 |
|  | O.flor (l) | O.flor (t) | 20.8 | 16.80 | 0.929 | -32.76 | 74.27 |
|  |  | O.sold | 121.4 | 14.88 | 0.000 | 70.71 | 172.04 |
|  |  | P.trin | 114.6 | 17.14 | 0.000 | 58.76 | 170.34 |
|  |  | N.stri | -121.4 | 26.84 | 0.000 | -200.15 | -42.73 |
|  | O.sold | O.flor (t) | -100.6 | 11.17 | 0.000 | -134.11 | -67.12 |
|  |  | O.flor (l) | -121.4 | 14.88 | 0.000 | -172.04 | -70.71 |
|  |  | P.trin | -6.8 | 11.68 | 1.000 | -46.98 | 33.33 |
|  |  | N.stri | -242.8 | 23.73 | 0.000 | -312.51 | -173.12 |
|  | P.trin | O.flor (t) | -93.8 | 14.05 | 0.000 | -138.07 | -49.52 |
|  |  | O.flor (l) | -114.6 | 17.14 | 0.000 | -170.34 | -58.76 |
|  |  | O.sold | 6.8 | 11.68 | 1.000 | -33.33 | 46.98 |
|  |  | N.stri | -236.0 | 25.21 | 0.000 | -309.90 | -162.09 |
|  | N.stri | O.flor (t) | 142.2 | 24.98 | 0.000 | 69.33 | 215.07 |
|  |  | O.flor (l) | 121.4 | 26.84 | 0.000 | 42.73 | 200.15 |
|  |  | O.sold | 242.8 | 23.73 | 0.000 | 173.12 | 312.51 |
|  |  | P.trin | 236.0 | 25.21 | 0.000 | 162.09 | 309.90 |
| DR | O.flor (t) | O.flor (l) | -0.2 | 0.06 | 0.039 | -0.46 | -0.01 |
|  |  | O.sold | -0.1 | 0.04 | 0.063 | -0.23 | 0.00 |
|  |  | P.trin | 0.1 | 0.05 | 0.288 | -0.06 | 0.32 |
|  |  | N.stri | 0.1 | 0.03 | 0.053 | 0.00 | 0.15 |
|  | O.flor (l) | O.flor (t) | 0.2 | 0.06 | 0.039 | 0.01 | 0.46 |
|  |  | O.sold | 0.1 | 0.07 | 0.643 | -0.11 | 0.36 |
|  |  | P.trin | 0.4 | 0.08 | 0.003 | 0.11 | 0.62 |
|  |  | N.stri | 0.3 | 0.07 | 0.006 | 0.09 | 0.54 |
|  | O.sold | O.flor (t) | 0.1 | 0.04 | 0.063 | 0.00 | 0.23 |
|  |  | O.flor (l) | -0.1 | 0.07 | 0.643 | -0.36 | 0.11 |
|  |  | P.trin | 0.2 | 0.06 | 0.011 | 0.05 | 0.44 |
|  |  | N.stri | 0.2 | 0.04 | 0.000 | 0.07 | 0.31 |
|  | P.trin | O.flor (t) | -0.1 | 0.05 | 0.288 | -0.32 | 0.06 |
|  |  | O.flor (l) | -0.4 | 0.08 | 0.003 | -0.62 | -0.11 |
|  |  | O.sold | -0.2 | 0.06 | 0.011 | -0.44 | -0.05 |
|  |  | N.stri | -0.1 | 0.05 | 0.984 | -0.24 | 0.14 |
|  | N.stri | O.flor (t) | -0.1 | 0.03 | 0.053 | -0.15 | 0.00 |
|  |  | O.flor (l) | -0.3 | 0.07 | 0.006 | -0.54 | -0.09 |
|  |  | O.sold | -0.2 | 0.04 | 0.000 | -0.31 | -0.07 |
|  |  | P.trin | 0.1 | 0.05 | 0.984 | -0.14 | 0.24 |
| IMR | O.flor (t) | O.flor (l) | -37.2 | 19.41 | 0.522 | -98.90 | 24.51 |
|  |  | O.sold | 138.5 | 14.08 | 0.000 | 96.75 | 180.23 |
|  |  | P.trin | 128.6 | 16.07 | 0.000 | 78.15 | 178.97 |
|  |  | N.stri | -104.9 | 24.76 | 0.001 | -176.90 | -32.85 |
|  | O.flor (l) | O.flor (t) | 37.2 | 19.41 | 0.522 | -24.51 | 98.90 |
|  |  | O.sold | 175.7 | 17.95 | 0.000 | 116.57 | 234.80 |
|  |  | P.trin | 165.8 | 19.55 | 0.000 | 102.05 | 229.45 |
|  |  | N.stri | -67.7 | 27.14 | 0.154 | -147.76 | 12.40 |
|  | O.sold | O.flor (t) | -138.5 | 14.08 | 0.000 | -180.23 | -96.75 |
|  |  | O.flor (l) | -175.7 | 17.95 | 0.000 | -234.80 | -116.57 |
|  |  | P.trin | -9.9 | 14.28 | 0.999 | -56.42 | 36.56 |
|  |  | N.stri | -243.4 | 23.63 | 0.000 | -312.36 | -174.37 |
|  | P.trin | O.flor (t) | -128.6 | 16.07 | 0.000 | -178.97 | -78.15 |
|  |  | O.flor (l) | -165.8 | 19.55 | 0.000 | -229.45 | -102.05 |
|  |  | O.sold | 9.9 | 14.28 | 0.999 | -36.56 | 56.42 |
|  |  | N.stri | -233.4 | 24.87 | 0.000 | -306.46 | -160.41 |
|  | N.stri | O.flor (t) | 104.9 | 24.76 | 0.001 | 32.85 | 176.90 |
|  |  | O.flor (l) | 67.7 | 27.14 | 0.154 | -12.40 | 147.76 |
|  |  | O.sold | 243.4 | 23.63 | 0.000 | 174.37 | 312.36 |
|  |  | P.trin | 233.4 | 24.87 | 0.000 | 160.41 | 306.46 |
| MRInc | O.flor (t) | O.flor (l) | 0.0 | 0.01 | 0.008 | -0.04 | -0.01 |
|  |  | O.sold | 0.0 | 0.00 | 0.001 | -0.03 | -0.01 |
|  |  | P.trin | 0.0 | 0.01 | 0.522 | -0.01 | 0.04 |
|  |  | N.stri | 0.1 | 0.00 | 0.000 | 0.04 | 0.06 |
|  | O.flor (l) | O.flor (t) | 0.0 | 0.01 | 0.008 | 0.01 | 0.04 |
|  |  | O.sold | 0.0 | 0.01 | 1.000 | -0.02 | 0.02 |
|  |  | P.trin | 0.0 | 0.01 | 0.003 | 0.01 | 0.06 |
|  |  | N.stri | 0.1 | 0.00 | 0.000 | 0.06 | 0.09 |
|  | O.sold | O.flor (t) | 0.0 | 0.00 | 0.001 | 0.01 | 0.03 |
|  |  | O.flor (l) | 0.0 | 0.01 | 1.000 | -0.02 | 0.02 |
|  |  | P.trin | 0.0 | 0.01 | 0.004 | 0.01 | 0.06 |
|  |  | N.stri | 0.1 | 0.00 | 0.000 | 0.06 | 0.08 |
|  | P.trin | O.flor (t) | 0.0 | 0.01 | 0.522 | -0.04 | 0.01 |
|  |  | O.flor (l) | 0.0 | 0.01 | 0.003 | -0.06 | -0.01 |
|  |  | O.sold | 0.0 | 0.01 | 0.004 | -0.06 | -0.01 |
|  |  | N.stri | 0.0 | 0.01 | 0.004 | 0.01 | 0.06 |
|  | N.stri | O.flor (t) | -0.1 | 0.00 | 0.000 | -0.06 | -0.04 |
|  |  | O.flor (l) | -0.1 | 0.00 | 0.000 | -0.09 | -0.06 |
|  |  | O.sold | -0.1 | 0.00 | 0.000 | -0.08 | -0.06 |
|  |  | P.trin | 0.0 | 0.01 | 0.004 | -0.06 | -0.01 |
| CBIn | O.flor (t) | O.flor (l) | 0.5 | 0.45 | 0.961 | -1.05 | 2.08 |
|  |  | O.sold | 1.7 | 0.23 | 0.000 | 0.98 | 2.35 |
|  |  | P.trin | 2.0 | 0.18 | 0.000 | 1.42 | 2.53 |
|  |  | N.stri | -0.1 | 0.27 | 1.000 | -0.91 | 0.64 |
|  | O.flor (l) | O.flor (t) | -0.5 | 0.45 | 0.961 | -2.08 | 1.05 |
|  |  | O.sold | 1.2 | 0.46 | 0.251 | -0.42 | 2.72 |
|  |  | P.trin | 1.5 | 0.44 | 0.075 | -0.11 | 3.03 |
|  |  | N.stri | -0.6 | 0.48 | 0.890 | -2.24 | 0.94 |
|  | O.sold | O.flor (t) | -1.7 | 0.23 | 0.000 | -2.35 | -0.98 |
|  |  | O.flor (l) | -1.2 | 0.46 | 0.251 | -2.72 | 0.42 |
|  |  | P.trin | 0.3 | 0.21 | 0.800 | -0.32 | 0.94 |
|  |  | N.stri | -1.8 | 0.29 | 0.000 | -2.63 | -0.97 |
|  | P.trin | O.flor (t) | -2.0 | 0.18 | 0.000 | -2.53 | -1.42 |
|  |  | O.flor (l) | -1.5 | 0.44 | 0.075 | -3.03 | 0.11 |
|  |  | O.sold | -0.3 | 0.21 | 0.800 | -0.94 | 0.32 |
|  |  | N.stri | -2.1 | 0.25 | 0.000 | -2.83 | -1.39 |
|  | N.stri | O.flor (t) | 0.1 | 0.27 | 1.000 | -0.64 | 0.91 |
|  |  | O.flor (l) | 0.6 | 0.48 | 0.890 | -0.94 | 2.24 |
|  |  | O.sold | 1.8 | 0.29 | 0.000 | 0.97 | 2.63 |
|  |  | P.trin | 2.1 | 0.25 | 0.000 | 1.39 | 2.83 |
| ICB | O.flor (t) | O.flor (l) | -15.9 | 11.78 | 0.892 | -55.15 | 23.36 |
|  |  | O.sold | 38.6 | 5.99 | 0.000 | 20.65 | 56.64 |
|  |  | P.trin | 41.6 | 8.16 | 0.001 | 15.50 | 67.79 |
|  |  | N.stri | -35.8 | 10.43 | 0.011 | -66.09 | -5.49 |
|  | O.flor (l) | O.flor (t) | 15.9 | 11.78 | 0.892 | -23.36 | 55.15 |
|  |  | O.sold | 54.5 | 10.95 | 0.005 | 15.87 | 93.21 |
|  |  | P.trin | 57.5 | 12.27 | 0.003 | 16.96 | 98.12 |
|  |  | N.stri | -19.9 | 13.89 | 0.835 | -62.65 | 22.86 |
|  | O.sold | O.flor (t) | -38.6 | 5.99 | 0.000 | -56.64 | -20.65 |
|  |  | O.flor (l) | -54.5 | 10.95 | 0.005 | -93.21 | -15.87 |
|  |  | P.trin | 3.0 | 6.91 | 1.000 | -21.49 | 27.49 |
|  |  | N.stri | -74.4 | 9.49 | 0.000 | -102.21 | -46.66 |
|  | P.trin | O.flor (t) | -41.6 | 8.16 | 0.001 | -67.79 | -15.50 |
|  |  | O.flor (l) | -57.5 | 12.27 | 0.003 | -98.12 | -16.96 |
|  |  | O.sold | -3.0 | 6.91 | 1.000 | -27.49 | 21.49 |
|  |  | N.stri | -77.4 | 10.98 | 0.000 | -110.05 | -44.82 |
|  | N.stri | O.flor (t) | 35.8 | 10.43 | 0.011 | 5.49 | 66.09 |
|  |  | O.flor (l) | 19.9 | 13.89 | 0.835 | -22.86 | 62.65 |
|  |  | O.sold | 74.4 | 9.49 | 0.000 | 46.66 | 102.21 |
|  |  | P.trin | 77.4 | 10.98 | 0.000 | 44.82 | 110.05 |
| BBA | O.flor (t) | O.flor (l) | -0.1 | 0.04 | 0.506 | -0.18 | 0.04 |
|  |  | O.sold | -0.1 | 0.03 | 0.001 | -0.21 | -0.04 |
|  |  | P.trin | 0.1 | 0.05 | 0.250 | -0.04 | 0.28 |
|  |  | N.stri | 0.4 | 0.02 | 0.000 | 0.32 | 0.47 |
|  | O.flor (l) | O.flor (t) | 0.1 | 0.04 | 0.506 | -0.04 | 0.18 |
|  |  | O.sold | -0.1 | 0.03 | 0.690 | -0.16 | 0.05 |
|  |  | P.trin | 0.2 | 0.05 | 0.024 | 0.02 | 0.35 |
|  |  | N.stri | 0.5 | 0.03 | 0.000 | 0.36 | 0.57 |
|  | O.sold | O.flor (t) | 0.1 | 0.03 | 0.001 | 0.04 | 0.21 |
|  |  | O.flor (l) | 0.1 | 0.03 | 0.690 | -0.05 | 0.16 |
|  |  | P.trin | 0.2 | 0.05 | 0.003 | 0.08 | 0.40 |
|  |  | N.stri | 0.5 | 0.02 | 0.000 | 0.46 | 0.58 |
|  | P.trin | O.flor (t) | -0.1 | 0.05 | 0.250 | -0.28 | 0.04 |
|  |  | O.flor (l) | -0.2 | 0.05 | 0.024 | -0.35 | -0.02 |
|  |  | O.sold | -0.2 | 0.05 | 0.003 | -0.40 | -0.08 |
|  |  | N.stri | 0.3 | 0.04 | 0.002 | 0.11 | 0.44 |
|  | N.stri | O.flor (t) | -0.4 | 0.02 | 0.000 | -0.47 | -0.32 |
|  |  | O.flor (l) | -0.5 | 0.03 | 0.000 | -0.57 | -0.36 |
|  |  | O.sold | -0.5 | 0.02 | 0.000 | -0.58 | -0.46 |
|  |  | P.trin | -0.3 | 0.04 | 0.002 | -0.44 | -0.11 |
| ICL | O.flor (t) | O.flor (l) | -147.3 | 37.28 | 0.014 | -270.76 | -23.75 |
|  |  | O.sold | 158.1 | 19.01 | 0.000 | 100.47 | 215.64 |
|  |  | P.trin | 135.3 | 19.60 | 0.000 | 75.53 | 195.02 |
|  |  | N.stri | 33.9 | 26.35 | 0.896 | -42.69 | 110.53 |
|  | O.flor (l) | O.flor (t) | 147.3 | 37.28 | 0.014 | 23.75 | 270.76 |
|  |  | O.sold | 305.3 | 34.09 | 0.000 | 184.06 | 426.55 |
|  |  | P.trin | 282.5 | 34.42 | 0.000 | 161.08 | 403.97 |
|  |  | N.stri | 181.2 | 38.66 | 0.002 | 56.22 | 306.13 |
|  | O.sold | O.flor (t) | -158.1 | 19.01 | 0.000 | -215.64 | -100.47 |
|  |  | O.flor (l) | -305.3 | 34.09 | 0.000 | -426.55 | -184.06 |
|  |  | P.trin | -22.8 | 12.50 | 0.586 | -62.42 | 16.86 |
|  |  | N.stri | -124.1 | 21.59 | 0.000 | -187.11 | -61.16 |
|  | P.trin | O.flor (t) | -135.3 | 19.60 | 0.000 | -195.02 | -75.53 |
|  |  | O.flor (l) | -282.5 | 34.42 | 0.000 | -403.97 | -161.08 |
|  |  | O.sold | 22.8 | 12.50 | 0.586 | -16.86 | 62.42 |
|  |  | N.stri | -101.4 | 22.11 | 0.000 | -166.21 | -36.50 |
|  | N.stri | O.flor (t) | -33.9 | 26.35 | 0.896 | -110.53 | 42.69 |
|  |  | O.flor (l) | -181.2 | 38.66 | 0.002 | -306.13 | -56.22 |
|  |  | O.sold | 124.1 | 21.59 | 0.000 | 61.16 | 187.11 |
|  |  | P.trin | 101.4 | 22.11 | 0.000 | 36.50 | 166.21 |
| CLInc | O.flor (t) | O.flor (l) | 0.0 | 0.01 | 0.873 | -0.03 | 0.01 |
|  |  | O.sold | 0.0 | 0.00 | 0.000 | -0.04 | -0.01 |
|  |  | P.trin | 0.0 | 0.01 | 0.865 | -0.01 | 0.03 |
|  |  | N.stri | 0.0 | 0.00 | 0.000 | 0.02 | 0.03 |
|  | O.flor (l) | O.flor (t) | 0.0 | 0.01 | 0.873 | -0.01 | 0.03 |
|  |  | O.sold | 0.0 | 0.01 | 0.184 | -0.04 | 0.00 |
|  |  | P.trin | 0.0 | 0.01 | 0.359 | -0.01 | 0.04 |
|  |  | N.stri | 0.0 | 0.01 | 0.003 | 0.01 | 0.05 |
|  | O.sold | O.flor (t) | 0.0 | 0.00 | 0.000 | 0.01 | 0.04 |
|  |  | O.flor (l) | 0.0 | 0.01 | 0.184 | 0.00 | 0.04 |
|  |  | P.trin | 0.0 | 0.01 | 0.001 | 0.01 | 0.06 |
|  |  | N.stri | 0.1 | 0.00 | 0.000 | 0.04 | 0.06 |
|  | P.trin | O.flor (t) | 0.0 | 0.01 | 0.865 | -0.03 | 0.01 |
|  |  | O.flor (l) | 0.0 | 0.01 | 0.359 | -0.04 | 0.01 |
|  |  | O.sold | 0.0 | 0.01 | 0.001 | -0.06 | -0.01 |
|  |  | N.stri | 0.0 | 0.01 | 0.188 | -0.01 | 0.04 |
|  | N.stri | O.flor (t) | 0.0 | 0.00 | 0.000 | -0.03 | -0.02 |
|  |  | O.flor (l) | 0.0 | 0.01 | 0.003 | -0.05 | -0.01 |
|  |  | O.sold | -0.1 | 0.00 | 0.000 | -0.06 | -0.04 |
|  |  | P.trin | 0.0 | 0.01 | 0.188 | -0.04 | 0.01 |
| PerR | O.flor (t) | O.flor (l) | -0.1 | 0.04 | 0.104 | -0.28 | 0.02 |
|  |  | O.sold | -0.1 | 0.02 | 0.000 | -0.21 | -0.08 |
|  |  | P.trin | 0.0 | 0.03 | 0.959 | -0.08 | 0.16 |
|  |  | N.stri | 0.2 | 0.02 | 0.000 | 0.14 | 0.26 |
|  | O.flor (l) | O.flor (t) | 0.1 | 0.04 | 0.104 | -0.02 | 0.28 |
|  |  | O.sold | 0.0 | 0.04 | 1.000 | -0.16 | 0.14 |
|  |  | P.trin | 0.2 | 0.05 | 0.039 | 0.01 | 0.34 |
|  |  | N.stri | 0.3 | 0.04 | 0.000 | 0.18 | 0.48 |
|  | O.sold | O.flor (t) | 0.1 | 0.02 | 0.000 | 0.08 | 0.21 |
|  |  | O.flor (l) | 0.0 | 0.04 | 1.000 | -0.14 | 0.16 |
|  |  | P.trin | 0.2 | 0.03 | 0.002 | 0.07 | 0.30 |
|  |  | N.stri | 0.3 | 0.02 | 0.000 | 0.29 | 0.40 |
|  | P.trin | O.flor (t) | 0.0 | 0.03 | 0.959 | -0.16 | 0.08 |
|  |  | O.flor (l) | -0.2 | 0.05 | 0.039 | -0.34 | -0.01 |
|  |  | O.sold | -0.2 | 0.03 | 0.002 | -0.30 | -0.07 |
|  |  | N.stri | 0.2 | 0.03 | 0.009 | 0.04 | 0.28 |
|  | N.stri | O.flor (t) | -0.2 | 0.02 | 0.000 | -0.26 | -0.14 |
|  |  | O.flor (l) | -0.3 | 0.04 | 0.000 | -0.48 | -0.18 |
|  |  | O.sold | -0.3 | 0.02 | 0.000 | -0.40 | -0.29 |
|  |  | P.trin | -0.2 | 0.03 | 0.009 | -0.28 | -0.04 |
| *. The mean difference is significant at the 0.05 level. | | | | | | | |
