# Supplementary material for: Morphometric analysis of Eocene nummulitids in western and central Cuba: taxonomy, biostratigraphy and evolutionary trends
Source: J Syst Palaeontol. 2018 Apr 13;17(7):557–95. doi: 10.1080/14772019.2018.1446462 (PMC6474738; doi:10.1080/14772019.2018.1446462)
Supplement: Anova_Genera.docx [file TJSP_A_1446462_SM8322.docx]

| **ANOVA genera** | | | | | | |
| --- | --- | --- | --- | --- | --- | --- |
|  |  | Sum of Squares | df | Mean Square | F | Sig. |
| FCL | Between Groups | 247756.7 | 2 | 123878.364 | 3.46 | 0.035 |
|  | Within Groups | 3397966.4 | 95 | 35768.067 |  |  |
|  | Total | 3645723.1 | 97 |  |  |  |
| PD | Between Groups | 873348.1 | 2 | 436674.054 | 34.88 | 0.000 |
|  | Within Groups | 1189283.6 | 95 | 12518.775 |  |  |
|  | Total | 2062631.7 | 97 |  |  |  |
| DR | Between Groups | 0.8 | 2 | .406 | 17.66 | 0.000 |
|  | Within Groups | 2.2 | 95 | .023 |  |  |
|  | Total | 3.0 | 97 |  |  |  |
| IMR | Between Groups | 684089.9 | 2 | 342044.939 | 26.05 | 0.000 |
|  | Within Groups | 1247183.1 | 95 | 13128.243 |  |  |
|  | Total | 1931272.9 | 97 |  |  |  |
| MRInc | Between Groups | 0.1 | 2 | .048 | 201.96 | 0.000 |
|  | Within Groups | 0.0 | 95 | .000 |  |  |
|  | Total | 0.1 | 97 |  |  |  |
| CBInc | Between Groups | 35.2 | 2 | 17.584 | 10.73 | 0.000 |
|  | Within Groups | 155.7 | 95 | 1.639 |  |  |
|  | Total | 190.9 | 97 |  |  |  |
| ICB | Between Groups | 59961.1 | 2 | 29980.574 | 14.56 | 0.000 |
|  | Within Groups | 195554.3 | 95 | 2058.467 |  |  |
|  | Total | 255515.5 | 97 |  |  |  |
| BBA | Between Groups | 5.2 | 2 | 2.596 | 308.45 | 0.000 |
|  | Within Groups | 0.8 | 95 | .008 |  |  |
|  | Total | 6.0 | 97 |  |  |  |
| ICL | Between Groups | 46646.3 | 2 | 23323.139 | 1.52 | 0.225 |
|  | Within Groups | 1461093.9 | 95 | 15379.935 |  |  |
|  | Total | 1507740.2 | 97 |  |  |  |
| CLInc | Between Groups | 0.0 | 2 | .018 | 61.57 | 0.000 |
|  | Within Groups | 0.0 | 95 | .000 |  |  |
|  | Total | 0.1 | 97 |  |  |  |
| PerR | Between Groups | 2.0 | 2 | .986 | 112.72 | 0.000 |
|  | Within Groups | 0.8 | 95 | .009 |  |  |
|  | Total | 2.8 | 97 |  |  |  |
| **Test of Homogeneity of Variances** | | | | |  |  |
|  | Levene Statistic | df1 | df2 | Sig. |  |  |
| FCL | 5.693 | 2 | 95 | .005 |  |  |
| PD | 37.220 | 2 | 95 | .000 |  |  |
| DR | 1.180 | 2 | 95 | .312 |  |  |
| IMR | 7.130 | 2 | 95 | .001 |  |  |
| MRInc | 10.614 | 2 | 95 | .000 |  |  |
| CBInc | 6.721 | 2 | 95 | .002 |  |  |
| ICB | 7.907 | 2 | 95 | .001 |  |  |
| BBA | 9.821 | 2 | 95 | .000 |  |  |
| ICL | 3.479 | 2 | 95 | .035 |  |  |
| CLInc | 15.717 | 2 | 95 | .000 |  |  |
| PerR | 6.667 | 2 | 95 | .002 |  |  |

| **Multiple Comparisons** | | | | | | | | |
| --- | --- | --- | --- | --- | --- | --- | --- | --- |
| Dependent Variable |  |  |  | Mean Difference (I-J) | SE | Sig. | 95% Confidence Interval |  |
|  |  |  |  |  |  |  | Lower Bound | Upper Bound |
| FCL | Tamhane | Oper. | Palaeon. | 125.7 | 30.00 | 0.002 | 45.59 | 205.85 |
|  |  |  | Nummulites | -79.8 | 41.06 | 0.158 | -180.06 | 20.51 |
|  |  | Palaeon. | Oper. | -125.7 | 30.00 | 0.002 | -205.85 | -45.59 |
|  |  |  | Nummulites | -205.5 | 39.11 | 0.000 | -304.46 | -106.54 |
|  |  | Nummulites | Oper. | 79.8 | 41.06 | 0.158 | -20.51 | 180.06 |
|  |  |  | Palaeon. | 205.5 | 39.11 | 0.000 | 106.54 | 304.46 |
| PD | Tamhane | Oper. | Palaeon. | 33.3 | 15.92 | 0.211 | -16.74 | 83.29 |
|  |  |  | Nummulites | -187.9 | 25.35 | 0.000 | -250.41 | -125.45 |
|  |  | Palaeon. | Oper. | -33.3 | 15.92 | 0.211 | -83.29 | 16.74 |
|  |  |  | Nummulites | -221.2 | 26.89 | 0.000 | -289.07 | -153.35 |
|  |  | Nummulites | Oper. | 187.9 | 25.35 | 0.000 | 125.45 | 250.41 |
|  |  |  | Palaeon. | 221.2 | 26.89 | 0.000 | 153.35 | 289.07 |
| DR | Tukey HSD | Oper. | Palaeon. | .27865^*^ | 0.08 | 0.002 | 0.09 | 0.47 |
|  |  |  | Nummulites | .16758^*^ | 0.03 | 0.000 | 0.09 | 0.24 |
|  |  | Palaeon. | Oper. | -0.3 | 0.08 | 0.002 | -0.47 | -0.09 |
|  |  |  | Nummulites | -0.1 | 0.08 | 0.345 | -0.30 | 0.08 |
|  |  | Nummulites | Oper. | -0.2 | 0.03 | 0.000 | -0.24 | -0.09 |
|  |  |  | Palaeon. | 0.1 | 0.08 | 0.345 | -0.08 | 0.30 |
| IMR | Tukey HSD | Oper. | Palaeon. | 51.9 | 59.45 | 0.658 | -89.64 | 193.47 |
|  |  |  | Nummulites | -163.9 | 23.77 | 0.000 | -220.48 | -107.28 |
|  |  | Palaeon. | Oper. | -51.9 | 59.45 | 0.658 | -193.47 | 89.64 |
|  |  |  | Nummulites | -215.8 | 59.96 | 0.001 | -358.55 | -73.05 |
|  |  | Nummulites | Oper. | 163.9 | 23.77 | 0.000 | 107.28 | 220.48 |
|  |  |  | Palaeon. | 215.8 | 59.96 | 0.001 | 73.05 | 358.55 |
| MRInc | Tamhane | Oper. | Palaeon. | 0.0 | 0.01 | 0.121 | -0.01 | 0.08 |
|  |  |  | Nummulites | 0.1 | 0.00 | 0.000 | 0.06 | 0.07 |
|  |  | Palaeon. | Oper. | 0.0 | 0.01 | 0.121 | -0.08 | 0.01 |
|  |  |  | Nummulites | 0.0 | 0.01 | 0.128 | -0.01 | 0.08 |
|  |  | Nummulites | Oper. | -0.1 | 0.00 | 0.000 | -0.07 | -0.06 |
|  |  |  | Palaeon. | 0.0 | 0.01 | 0.128 | -0.08 | 0.01 |
| Cbinc | Tamhane | Oper. | Palaeon. | 1.0 | 0.16 | 0.000 | 0.63 | 1.43 |
|  |  |  | Nummulites | -1.1 | 0.28 | 0.001 | -1.74 | -0.39 |
|  |  | Palaeon. | Oper. | -1.0 | 0.16 | 0.000 | -1.43 | -0.63 |
|  |  |  | Nummulites | -2.1 | 0.23 | 0.000 | -2.67 | -1.52 |
|  |  | Nummulites | Oper. | 1.1 | 0.28 | 0.001 | 0.39 | 1.74 |
|  |  |  | Palaeon. | 2.1 | 0.23 | 0.000 | 1.52 | 2.67 |
| ICB | Tamhane | Oper. | Palaeon. | 16.0 | 6.77 | 0.117 | -3.51 | 35.51 |
|  |  |  | Nummulites | -48.4 | 10.11 | 0.000 | -73.26 | -23.63 |
|  |  | Palaeon. | Oper. | -16.0 | 6.77 | 0.117 | -35.51 | 3.51 |
|  |  |  | Nummulites | -64.4 | 10.32 | 0.000 | -90.52 | -38.38 |
|  |  | Nummulites | Oper. | 48.4 | 10.11 | 0.000 | 23.63 | 73.26 |
|  |  |  | Palaeon. | 64.4 | 10.32 | 0.000 | 38.38 | 90.52 |
| BBA | Tamhane | Oper. | Palaeon. | 0.1 | 0.07 | 0.317 | -0.16 | 0.44 |
|  |  |  | Nummulites | 0.5 | 0.02 | 0.000 | 0.43 | 0.51 |
|  |  | Palaeon. | Oper. | -0.1 | 0.07 | 0.317 | -0.44 | 0.16 |
|  |  |  | Nummulites | 0.3 | 0.07 | 0.043 | 0.02 | 0.64 |
|  |  | Nummulites | Oper. | -0.5 | 0.02 | 0.000 | -0.51 | -0.43 |
|  |  |  | Palaeon. | -0.3 | 0.07 | 0.043 | -0.64 | -0.02 |
| ICL | Tamhane | Oper. | Palaeon. | 85.0 | 19.54 | 0.000 | 36.32 | 133.74 |
|  |  |  | Nummulites | -22.6 | 26.11 | 0.771 | -86.18 | 40.89 |
|  |  | Palaeon. | Oper. | -85.0 | 19.54 | 0.000 | -133.74 | -36.32 |
|  |  |  | Nummulites | -107.7 | 21.14 | 0.000 | -160.45 | -54.89 |
|  |  | Nummulites | Oper. | 22.6 | 26.11 | 0.771 | -40.89 | 86.18 |
|  |  |  | Palaeon. | 107.7 | 21.14 | 0.000 | 54.89 | 160.45 |
| CLInc | Tamhane | Oper. | Palaeon. | 0.0 | 0.01 | 0.274 | -0.02 | 0.06 |
|  |  |  | Nummulites | 0.0 | 0.00 | 0.000 | 0.03 | 0.05 |
|  |  | Palaeon. | Oper. | 0.0 | 0.01 | 0.274 | -0.06 | 0.02 |
|  |  |  | Nummulites | 0.0 | 0.01 | 0.315 | -0.02 | 0.06 |
|  |  | Nummulites | Oper. | 0.0 | 0.00 | 0.000 | -0.05 | -0.03 |
|  |  |  | Palaeon. | 0.0 | 0.01 | 0.315 | -0.06 | 0.02 |
| PerR | Tamhane | Oper. | Palaeon. | 0.1 | 0.06 | 0.314 | -0.12 | 0.35 |
|  |  |  | Nummulites | 0.3 | 0.02 | 0.000 | 0.25 | 0.34 |
|  |  | Palaeon. | Oper. | -0.1 | 0.06 | 0.314 | -0.35 | 0.12 |
|  |  |  | Nummulites | 0.2 | 0.05 | 0.128 | -0.08 | 0.43 |
|  |  | Nummulites | Oper. | -0.3 | 0.02 | 0.000 | -0.34 | -0.25 |
|  |  |  | Palaeon. | -0.2 | 0.05 | 0.128 | -0.43 | 0.08 |
