# Supplementary material for: Morphometric analysis of Eocene nummulitids in western and central Cuba: taxonomy, biostratigraphy and evolutionary trends
Source: J Syst Palaeontol. 2018 Apr 13;17(7):557–95. doi: 10.1080/14772019.2018.1446462 (PMC6474738; doi:10.1080/14772019.2018.1446462)
Supplement: CDA_and_PC_loadings.docx [file TJSP_A_1446462_SM8319.docx]

| **All specimen** | | | | | | |
| --- | --- | --- | --- | --- | --- | --- |
| **Structure Matrix** | | | | | | |
|  | Function | | | | | |
|  | 1 | 2 | 3 | 4 | 5 | 6 |
| BBA | .604^*^ | .389 | .227 | .124 | -.483 | -.108 |
| MRInc | .543^*^ | .273 | -.059 | .045 | .169 | .254 |
| PerR | .454^*^ | .352 | .358 | .236 | .101 | -.372 |
| FCL | -.300 | .752^*^ | .144 | .070 | .156 | -.244 |
| ICL | -.210 | .645^*^ | -.189 | -.073 | .086 | -.214 |
| PD | -.440 | .273 | .551^*^ | -.008 | -.192 | -.118 |
| IMR | -.531 | .483 | -.112 | .605^*^ | -.117 | -.067 |
| CLInc | .356 | .070 | .140 | .389^*^ | .147 | .304 |
| ICB | -.333 | .350 | .164 | .371^*^ | -.138 | -.250 |
| DR | .138 | .130 | .173 | -.105 | .501 | .581^*^ |
| CBInc | -.183 | .191 | -.126 | .165 | -.377 | .440^*^ |
| Pooled within-groups correlations between discriminating variables and standardized canonical discriminant functions   Variables ordered by absolute size of correlation within function. | | | | | | |
| *. Largest absolute correlation between each variable and any discriminant function | | | | | | |
| **Eigenvalues** | | | | |  |  |
| Function | Eigenvalue | % of Variance | Cumulative % | Canonical Correlation |  |  |
| 1 | 17.479^a^ | 64.9 | 64.9 | .973 |  |  |
| 2 | 6.482^a^ | 24.1 | 89.0 | .931 |  |  |
| 3 | 1.643^a^ | 6.1 | 95.1 | .788 |  |  |
| 4 | .745^a^ | 2.8 | 97.8 | .653 |  |  |
| 5 | .363^a^ | 1.3 | 99.2 | .516 |  |  |
| 6 | .222^a^ | .8 | 100.0 | .426 |  |  |
| a. First 6 canonical discriminant functions were used in the analysis. | | | | |  |  |

| **Operculinoides** | | | | |
| --- | --- | --- | --- | --- |
| **Structure Matrix** | | | |  |
|  | Function | | |  |
|  | 1 | 2 | 3 |  |
| ICL | .642^*^ | .310 | -.077 |  |
| IMR | .611^*^ | .063 | .141 |  |
| PD | .574^*^ | .063 | .058 |  |
| FCL | .539^*^ | .474 | -.208 |  |
| ICB | .449^*^ | .190 | -.103 |  |
| MRInc | -.104 | .526^*^ | .315 |  |
| PerR | -.186 | .505^*^ | .072 |  |
| DR | -.063 | .475^*^ | .325 |  |
| CLInc | -.236 | .349^*^ | .228 |  |
| BBA | -.161 | .346^*^ | .330 |  |
| CBInc | .330 | -.197 | .693^*^ |  |
| Pooled within-groups correlations between discriminating variables and standardized canonical discriminant functions   Variables ordered by absolute size of correlation within function. | | | |  |
| *. Largest absolute correlation between each variable and any discriminant function | | | |  |
| **Eigenvalues** | | | | |
| Function | Eigenvalue | % of Variance | Cumulative % | Canonical Correlation |
| 1 | 6.673^a^ | 76.2 | 76.2 | .933 |
| 2 | 1.672^a^ | 19.1 | 95.3 | .791 |
| 3 | .412^a^ | 4.7 | 100.0 | .540 |
| a. First 3 canonical discriminant functions were used in the analysis. | | | | |

| **Nummulites** | | | | |
| --- | --- | --- | --- | --- |
| **Structure Matrix** | | | |  |
|  | Function | | |  |
|  | 1 | 2 | 3 |  |
| FCL | .546^*^ | .299 | -.137 |  |
| IMR | .396^*^ | .011 | -.306 |  |
| ICB | .284^*^ | .099 | .245 |  |
| CBInc | .257^*^ | -.110 | .203 |  |
| PD | .239 | .505^*^ | -.156 |  |
| PerR | .186 | .285^*^ | .270 |  |
| BBA | .073 | .320 | .523^*^ |  |
| ICL | .433 | .136 | -.519^*^ |  |
| MRInc | -.117 | -.174 | .237^*^ |  |
| CLInc | -.098 | -.162 | .232^*^ |  |
| DR | -.098 | .108 | .161^*^ |  |
| Pooled within-groups correlations between discriminating variables and standardized canonical discriminant functions   Variables ordered by absolute size of correlation within function. | | | |  |
| *. Largest absolute correlation between each variable and any discriminant function | | | |  |
|  |  |  |  |  |
|  |  |  |  |  |
| **Eigenvalues** | | | | |
| Function | Eigenvalue | % of Variance | Cumulative % | Canonical Correlation |
| 1 | 29.618^a^ | 63.9 | 63.9 | .984 |
| 2 | 15.706^a^ | 33.9 | 97.7 | .970 |
| 3 | 1.051^a^ | 2.3 | 100.0 | .716 |
| a. First 3 canonical discriminant functions were used in the analysis. | | | | |

| ***O. floridensis*** | | | | |
| --- | --- | --- | --- | --- |
| **Structure Matrix** | | |  |  |
|  | Function | |  |  |
|  | 1 | 2 |  |  |
| PerR | .692^*^ | .080 |  |  |
| FCL | .462^*^ | .355 |  |  |
| MRInc | .317^*^ | -.156 |  |  |
| ICL | .187^*^ | .132 |  |  |
| BBA | .165^*^ | -.077 |  |  |
| DR | .120^*^ | -.086 |  |  |
| ICB | .138 | .541^*^ |  |  |
| PD | .051 | .265^*^ |  |  |
| IMR | .018 | .236^*^ |  |  |
| CBInc | -.165 | .189^*^ |  |  |
| CLInc | .021 | -.072^*^ |  |  |
| Pooled within-groups correlations between discriminating variables and standardized canonical discriminant functions   Variables ordered by absolute size of correlation within function. | | |  |  |
| *. Largest absolute correlation between each variable and any discriminant function | | |  |  |
| **Eigenvalues** | | | | |
| Function | Eigenvalue | % of Variance | Cumulative % | Canonical Correlation |
| 1 | 2.791^a^ | 54.5 | 54.5 | .858 |
| 2 | 2.326^a^ | 45.5 | 100.0 | .836 |
| a. First 2 canonical discriminant functions were used in the analysis. | | | | |

| ***O. soldadensis*** | | | | |
| --- | --- | --- | --- | --- |
| **Structure Matrix** | | |  |  |
|  | Function | |  |  |
|  | 1 | 2 |  |  |
| CBInc | .299^*^ | .247 |  |  |
| DR | .179^*^ | .045 |  |  |
| PerR | -.048^*^ | -.009 |  |  |
| ICB | .021^*^ | -.001 |  |  |
| MRInc | -.009 | -.564^*^ |  |  |
| CLInc | .097 | -.527^*^ |  |  |
| ICL | -.005 | .430^*^ |  |  |
| BBA | .079 | .195^*^ |  |  |
| FCL | .003 | .133^*^ |  |  |
| IMR | .074 | .104^*^ |  |  |
| PD | -.054 | .088^*^ |  |  |
| Pooled within-groups correlations between discriminating variables and standardized canonical discriminant functions   Variables ordered by absolute size of correlation within function. | | |  |  |
| *. Largest absolute correlation between each variable and any discriminant function | | |  |  |
|  |  |  |  |  |
|  |  |  |  |  |
| **Eigenvalues** | | | | |
| Function | Eigenvalue | % of Variance | Cumulative % | Canonical Correlation |
| 1 | 8.863^a^ | 85.2 | 85.2 | .948 |
| 2 | 1.541^a^ | 14.8 | 100.0 | .779 |
| a. First 2 canonical discriminant functions were used in the analysis. | | | | |

| **Operculinoides + Heterostegina** | | | | | |
| --- | --- | --- | --- | --- | --- |
| **Structure Matrix** | | | | | |
|  | Function | | | | |
|  | 1 | 2 | 3 | 4 | 5 |
| PerR | .667^*^ | -.062 | .050 | .430 | -.383 |
| BBA | .502^*^ | -.054 | -.019 | -.143 | .082 |
| ICL | .426 | .676^*^ | -.033 | .162 | .464 |
| IMR | -.016 | .661^*^ | -.056 | .026 | .229 |
| PD | .109 | .636^*^ | -.112 | .000 | .190 |
| ICB | .080 | .480^*^ | -.035 | -.034 | .404 |
| FCL | -.011 | .471^*^ | .251 | .426 | .281 |
| MRI | .368 | -.109 | .589^*^ | -.035 | -.151 |
| CBI | -.162 | .367 | -.522^*^ | -.028 | -.055 |
| DR | .112 | -.096 | .401^*^ | .291 | -.120 |
| CLI | .035 | -.286 | .143 | -.008 | .292^*^ |
| Pooled within-groups correlations between discriminating variables and standardized canonical discriminant functions   Variables ordered by absolute size of correlation within function. | | | | | |
| *. Largest absolute correlation between each variable and any discriminant function | | | | | |
|  |  |  |  |  |  |
| **Eigenvalues** | | | | |  |
| Function | Eigenvalue | % of Variance | Cumulative % | Canonical Correlation |  |
| 1 | 12.012^a^ | 61.3 | 61.3 | .961 |  |
| 2 | 5.074^a^ | 25.9 | 87.2 | .914 |  |
| 3 | 1.333^a^ | 6.8 | 94.0 | .756 |  |
| 4 | .774^a^ | 3.9 | 98.0 | .660 |  |
| 5 | .396^a^ | 2.0 | 100.0 | .533 |  |
| a. First 5 canonical discriminant functions were used in the analysis. | | | | |  |

| Eigenvalues Figure 3 | | |
| --- | --- | --- |
| PC | Eigenvalue | % variance |
| 1 | 6.10 | 55.43 |
| 2 | 2.44 | 22.19 |
| 3 | 0.73 | 6.64 |
| 4 | 0.66 | 5.96 |
| 5 | 0.39 | 3.56 |
| 6 | 0.24 | 2.18 |
| 7 | 0.15 | 1.39 |
| 8 | 0.12 | 1.06 |
| 9 | 0.08 | 0.75 |
| 10 | 0.06 | 0.55 |
| 11 | 0.03 | 0.31 |

| Eigenvalues Figure 5 | | |
| --- | --- | --- |
| PC | Eigenvalue | % variance |
| 1 | 4.30 | 39.01 |
| 2 | 2.99 | 27.10 |
| 3 | 1.13 | 10.25 |
| 4 | 0.91 | 8.23 |
| 5 | 0.70 | 6.39 |
| 6 | 0.28 | 2.52 |
| 7 | 0.24 | 2.19 |
| 8 | 0.20 | 1.84 |
| 9 | 0.12 | 1.09 |
| 10 | 0.10 | 0.94 |
| 11 | 0.05 | 0.45 |
