# Supplementary material for: Morphometric analysis of Eocene nummulitids in western and central Cuba: taxonomy, biostratigraphy and evolutionary trends
Source: J Syst Palaeontol. 2018 Apr 13;17(7):557–95. doi: 10.1080/14772019.2018.1446462 (PMC6474738; doi:10.1080/14772019.2018.1446462)
Supplement: repository.docx [file TJSP_A_1446462_SM8313.docx]

| Specimen | assigned species | Sample | Locality |
| --- | --- | --- | --- |
| 98LC-1H(626) | *N. striatoreticulatus* | 98LC-1H | 98LC-1 |
| 98LC-1H(627) | *N. striatoreticulatus* | 98LC-1H | 98LC-1 |
| 98LC-1H(628) | *N. striatoreticulatus* | 98LC-1H | 98LC-1 |
| 98LC-1H(630) | *N. striatoreticulatus* | 98LC-1H | 98LC-1 |
| 98LC-1H(631) | *N. striatoreticulatus* | 98LC-1H | 98LC-1 |
| 98LC-1H(633) | *N. striatoreticulatus* | 98LC-1H | 98LC-1 |
| 98LC-1H(634) | *N. striatoreticulatus* | 98LC-1H | 98LC-1 |
| 98LC-1H(635) | *N. striatoreticulatus* | 98LC-1H | 98LC-1 |
| 98LC-1H(650) | *N. striatoreticulatus* | 98LC-1H | 98LC-1 |
| 98LC-1H(806) | *N. striatoreticulatus* | 98LC-1H | 98LC-1 |
| 98LC-1H(812) | *N. striatoreticulatus* | 98LC-1H | 98LC-1 |
| 98LC1h-622 | *O.floridensis (tight)* | 98LC-1H | 98LC-1 |
| 98LC1h-623 | *O.floridensis (loose)* | 98LC-1H | 98LC-1 |
| 98LC1h-624 | *O.floridensis (loose)* | 98LC-1H | 98LC-1 |
| 98LC1h-648 | *O.floridensis (tight)* | 98LC-1H | 98LC-1 |
| 98LC1h-651 | *O.floridensis (tight)* | 98LC-1H | 98LC-1 |
| 98LC1h-676 | *P.trinitatensis* | 98LC-1H | 98LC-1 |
| 98LC1h-815a | *O.floridensis (tight)* | 98LC-1H | 98LC-1 |
| 98LC1h-815b | *O.floridensis (tight)* | 98LC-1H | 98LC-1 |
| 98LC1h-815c | *O.floridensis (tight)* | 98LC-1H | 98LC-1 |
| 98LC1h-816 | *O.floridensis (tight)* | 98LC-1H | 98LC-1 |
| 98LC1h-817 | *O.floridensis (loose)* | 98LC-1H | 98LC-1 |
| 98LC1h-817b | *O.floridensis (tight)* | 98LC-1H | 98LC-1 |
| 98LC1h-CT11 | *P.trinitatensis* | 98LC-1H | 98LC-1 |
| 98LC1h-CT13 | *O.floridensis (tight)* | 98LC-1H | 98LC-1 |
| 98LC1h-CT15 | *O.floridensis (tight)* | 98LC-1H | 98LC-1 |
| 98LC1h-CT16 | *O.floridensis (tight)* | 98LC-1H | 98LC-1 |
| 98LC1h-CT20 | *P.trinitatensis* | 98LC-1H | 98LC-1 |
| 98LC1h-CT22 | *O.floridensis (tight)* | 98LC-1H | 98LC-1 |
| 98LC1h-CT3 | *O.floridensis (tight)* | 98LC-1H | 98LC-1 |
| 98LC1h-CT5 | *O.soldadensis* | 98LC-1H | 98LC-1 |
| 98LC1h-CT7 | *O.soldadensis* | 98LC-1H | 98LC-1 |
| 98LC1h-CT8 | *O.floridensis (tight)* | 98LC-1H | 98LC-1 |
| 98LC1i-666 | *O.soldadensis* | 98LC-1I | 98LC-1 |
| 98LC1i-667 | *O.floridensis (tight)* | 98LC-1I | 98LC-1 |
| 98LC1i-669 | *O.soldadensis* | 98LC-1I | 98LC-1 |
| 98LC1i-670 | *O.floridensis (tight)* | 98LC-1I | 98LC-1 |
| 98LC1i-CT3 | *P.trinitatensis* | 98LC-1I | 98LC-1 |
| 98LC1i-CT4 | *O.soldadensis* | 98LC-1I | 98LC-1 |
| 98LC2-1 | *N. striatoreticulatus* | 98LC-2 | 98LC-2 |
| 98LC2-11 | *N. striatoreticulatus* | 98LC-2 | 98LC-2 |
| 98LC2-12 | *N. striatoreticulatus* | 98LC-2 | 98LC-2 |
| 98LC2-13 | *N. striatoreticulatus* | 98LC-2 | 98LC-2 |
| 98LC2-15 | *N. striatoreticulatus* | 98LC-2 | 98LC-2 |
| 98LC2-2 | *N. striatoreticulatus* | 98LC-2 | 98LC-2 |
| 98LC2-3 | *N. striatoreticulatus* | 98LC-2 | 98LC-2 |
| 98LC2-6 | *N. striatoreticulatus* | 98LC-2 | 98LC-2 |
| 98LC2-686 | *N. striatoreticulatus* | 98LC-2 | 98LC-2 |
| 98LC2-687 | *N. striatoreticulatus* | 98LC-2 | 98LC-2 |
| 98LC2-689 | *N. striatoreticulatus* | 98LC-2 | 98LC-2 |
| 98LC2-690 | *N. striatoreticulatus* | 98LC-2 | 98LC-2 |
| 98LC2-691 | *N. striatoreticulatus* | 98LC-2 | 98LC-2 |
| 98LC2-692 | *N. striatoreticulatus* | 98LC-2 | 98LC-2 |
| 98LC2-7 | *N. striatoreticulatus* | 98LC-2 | 98LC-2 |
| CA-215(49) | *O.soldadensis* | CA-215 | CA-215 |
| CA-215(65) | *N. striatoreticulatus* | CA-215 | CA-215 |
| CA-215(852) | *O.floridensis (tight)* | CA-215 | CA-215 |
| CA-215(853) | *O.floridensis (loose)* | CA-215 | CA-215 |
| CA-215(862) | *N. striatoreticulatus* | CA-215 | CA-215 |
| CA-215(863) | *N. striatoreticulatus* | CA-215 | CA-215 |
| CA-215(865) | *N. striatoreticulatus* | CA-215 | CA-215 |
| CA-215(866) | *N. striatoreticulatus* | CA-215 | CA-215 |
| CA-215(867) | *N. striatoreticulatus* | CA215-17 | CA-215 |
| CA-215(868) | *N. striatoreticulatus* | CA215-17 | CA-215 |
| CA-215(872) | *O.floridensis (tight)* | CA215-17 | CA-215 |
| CA-215-17(55) | *O.soldadensis* | CA-215 | CA-215 |
| CA-215-17(63) | *O.soldadensis* | CA-215 | CA-215 |
| CA-215-17(875) | *O.soldadensis* | CA-215 | CA-215 |
| CA-216-F3(16) | *O.floridensis (loose)* | CA216-F3 | CA-216 |
| CA-216-F3(823a) | *O.floridensis (loose)* | CA216-F3 | CA-216 |
| CA-216-F3(823c) | *O.floridensis (tight)* | CA216-F3 | CA-216 |
| CA-216-F3(824a) | *O.floridensis (loose)* | CA216-F3 | CA-216 |
| E-126(458) | *N. striatoreticulatus* | E-126 | E-126 |
| E-126(459c) | *N. striatoreticulatus* | E-126 | E-126 |
| E-126(461b) | *N. striatoreticulatus* | E-126 | E-126 |
| E-126(462b) | *N. striatoreticulatus* | E-126 | E-126 |
| E-126(463b) | *N. striatoreticulatus* | E-126 | E-126 |
| E-126(464a) | *N. striatoreticulatus* | E-126 | E-126 |
| E-126(464b) | *N. striatoreticulatus* | E-126 | E-126 |
| E-126(465a) | *N. striatoreticulatus* | E-126 | E-126 |
| E-126(466a) | *N. striatoreticulatus* | E-126 | E-126 |
| CA-4(727) | *O.soldadensis* | LM-52 | LM-52 |
| LM-52(759) | *O.floridensis (tight)* | LM-52 | LM-52 |
| Nor14-1 | *O.soldadensis* | NOR-UN14/15 | NOR-UN |
| Nor14-1A | *O.soldadensis* | NOR-UN14/15 | NOR-UN |
| Nor14-2A | *O.soldadensis* | NOR-UN14/15 | NOR-UN |
| Nor14-3 | *O.soldadensis* | NOR-UN14/15 | NOR-UN |
| Nor14-4 | *O.soldadensis* | NOR-UN14/15 | NOR-UN |
| Nor14-6 | *O.soldadensis* | NOR-UN14/15 | NOR-UN |
| Nor14-7 | *O.soldadensis* | NOR-UN14/15 | NOR-UN |
| Nor14-8 | *O.soldadensis* | NOR-UN14/15 | NOR-UN |
| Nor14-9 | *O.soldadensis* | NOR-UN14/15 | NOR-UN |
| Nor14-r5 | *O.soldadensis* | NOR-UN14/15 | NOR-UN |
| Nor14-r6 | *O.soldadensis* | NOR-UN14/15 | NOR-UN |
| Nor14-r7.1 | *O.soldadensis* | NOR-UN14/15 | NOR-UN |
| Nor24-11 | *O.soldadensis* | NOR-UN24/25 | NOR-UN |
| Nor24-12 | *O.soldadensis* | NOR-UN24/25 | NOR-UN |
| Nor24-6 | *O.soldadensis* | NOR-UN24/25 | NOR-UN |
| *O. kugleri* | *P.trinitatensis* | Vaughan & Cole '41 (H) | Type |
| *O. spiralis* | *P.trinitatensis* | Caudri '75 (H) | Type |
| *O. trinitatentsis* | *P.trinitatensis* | Vaughan & Cole '41 (H) | Type |
| *O. willcoxi* | *P.trinitatensis?* | Barker '39 | Type |
| *O. willcoxi* | *O.floridensis (tight)* | Cole '41 | Type |
| *O.floridensis* | *O.floridensis (loose)* | Cole '41 | Type |
| *O.floridensis* | *O.floridensis (loose)* | Cole '41 | Type |
| *O.floridensis* | *O.floridensis (loose)* | Frost ' 96 | Type |
| *O.soldadensis* | *O.soldadensis* | Vaughan & Cole '41 (H) | Type |
| *O.suteri* | *O.soldadensis* | Caudri '96 | Type |
| *N. floridensis* | *O.soldadensis* | Butterlin '61 | Type |
| *N. macgillavry* | *N. macgillavry* | ? | Type |
| *N. striatoreticulatus* | *N. striatoreticulatus* | Rutten '28 (H) | Type |
| *N. trinitatensis* | P.trinitatensis | Butterlin '61 | Type |
